# Customer churn prediction model based on hybrid neural networks

**DOI:** 10.1038/s41598-024-79603-9

**Published:** 2024-12-28

**Authors:** Xinyu Liu, Guoen Xia, Xianquan Zhang, Wenbin Ma, Chunqiang Yu

**Affiliations:** 1 College of Computer Science and Engineering, Guangxi Normal University, Guilin, 541000 China; 2 College of Business Administration, Guangxi University, Nanning, 530000 China; 3 College of Business Administration, Guangxi University of Finance and Economics, Nanning, 530000 China

**Keywords:** Hybrid neural network, Churn prediction, Deep learning, Computer science, Information technology

## Abstract

In today’s competitive market environment, accurately identifying potential churn customers and taking effective retention measures are crucial for improving customer retention and ensuring the sustainable development of an organization. However, traditional machine learning algorithms and single deep learning models have limitations in extracting complex nonlinear and time-series features, resulting in unsatisfactory prediction results. To address this problem, this study proposes a hybrid neural network-based customer churn prediction model, CCP-Net. In the data preprocessing stage, the ADASYN sampling algorithm balances the sample sizes of churned and non-churned customers to eliminate the negative impact of sample imbalance on the model performance. In the feature extraction stage, CCP-Net uses Multi-Head Self-Attention to learn the global dependencies of the input sequences, combines with BiLSTM to capture the long-term dependencies in the sequential data, and uses CNN to extract the local features, and ultimately generates the prediction results. Experimental results of cross-validation on Telecom, Bank, Insurance, and News datasets show that CCP-Net outperforms the comparison algorithms in all performance metrics. For example, CCP-Net achieves a Precision of 92.19% on the Telecom dataset, 91.96% on the Bank dataset, 95.87% on the Insurance dataset, and 95.12% on the News dataset, which compares to other hybrid neural network models, the performance improvement of CCP-Net ranges from 1% to 3%. These results indicate that the design of the CCP-Net model effectively improves the accuracy and robustness of churn prediction, enabling it to be widely applied to different industries, especially in the financial, telecommunication, and media fields, to provide more comprehensive and effective churn management strategies for enterprises.

## Introduction

In today’s increasingly competitive business environment, the impact of customer churn on organizations is becoming increasingly significant. Churn refers to the behavior of customers who stop purchasing or using a company’s products or services, including canceling subscriptions, terminating services, or switching to competitors. This phenomenon directly cuts down an organization’s revenue and may damage its reputation, thus threatening its long-term development.

With the advent of the big data era, the application of Customer Relationship Management (CRM) systems in customer data analysis is becoming increasingly critical. CRM systems can integrate customer contact information, interaction records, and purchase history, helping organizations comprehensively understand customers’ needs and gain real-time access to customer trends^1^. Studies have shown that increased customer churn significantly reduces business profits, and the cost of acquiring new customers is usually five times higher than maintaining existing ones^2,3^. Therefore, in the fierce market competition, enterprises need to carry out customer churn prediction, identify potential lost customers, and take targeted retention strategies to improve customer loyalty and ensure the sustainable development of enterprises^4^.

## Related work

### Literature review

#### Machine learning algorithm

In the field of customer churn prediction, traditional methods mainly rely on machine learning algorithms, including Support Vector Machine(SVM)^1,5^, K-means clustering algorithm (K-means), Logistic Regression (LR)^6,7^, Naive Bayes (NB)^6,8^, K-nearest Neighbor (KNN), Decision Tree (DT)^7–9^, Random Forest (RF)^7,8^, LGBM, Adaboost^10^, and XGBoost^11^.

Xiahou et al.^1^ proposed using K-means combined with SVM for customer churn prediction. The study showed that customer clustering by K-means significantly improves prediction accuracy. NV et al.^6^ explored the application of algorithms such as LR and NB in banking datasets and found that NB outperforms LR. Kiguchi et al.^7^ investigated the predictive effectiveness of LR, DT, and RF in the education market and the results showed that LR has the best performance. Ahmad et al.^9^ tested algorithms such as DT, RF, and XGBoost in the Spark environment and found that XGBoost performs best. Lalwani et al.^10^ performed feature selection by gravitational search algorithms and found that AdaBoost and XGBoost are the leading performances among multiple models. Sikri et al.^12^ proposed a Ratio-based sample balancing technique for unbalanced customer churn data and combined it with multiple independent machine learning algorithms for prediction. Dhanawade et al.^11^ significantly improved the model’s performance for minority-class customer identification by combining multiple machine learning algorithms with SMOTE. The model can identify customers in a few categories and its prediction accuracy. He et al.^13^ proposed an integrated learning approach called Ensemble-Fusion, which combines 17 algorithms and outperforms a single model.

Although traditional machine learning algorithms perform well in processing structured data and simple feature engineering tasks, they are limited in extracting complex nonlinear features and processing large-scale datasets as the size and complexity of the data increase, leading to a decrease in prediction accuracy.

#### Single deep learning algorithm

In contrast to machine learning algorithms, deep learning algorithms show significant advantages in dealing with high-dimensional data and complex feature relationships, so more and more researchers are beginning to apply deep learning to customer churn prediction. Common deep learning algorithms include Backpropagation (BP)^14^, Artificial Neural Networks (ANN)^15,16^, Multi-Layer Perceptron (MLP)^17,18^, Convolutional Neural Network (CNN)^19^, Recurrent Neural Network (RNN)^20^, Long Short-Term Memory (LSTM)^19,20^, Bi-directional Long Short-Term Memory (BiLSTM)^21^, TabNet^22^,and Transformer^23^.

Khine et al.^24^ proposed a model combining K-means and MLP for bank customer churn prediction, which performs user clustering through K-means and subsequently uses MLP for prediction, and the results show that the model has high prediction accuracy and short training time. Venkatesh et al.^25^ developed an MLP model based on Artificial Fish Swarm Algorithm for customer churn prediction in IoT environment with an accuracy of 93.52%. In addition, Saha et al.^15^ tested integrated learning algorithms (AdaBoost, RF, XGBoost, LGBM) with ANNs and CNNs and found that ANNs and CNNs exhibited the highest accuracy in the two datasets, respectively. Zhang et al.^21^ explored the application of BiLSTM for customer churn prediction, and the experiments showed that BiLSTM outperforms traditional machine learning methods in terms of accuracy. Latheef et al.^26^ proposed the use of SMOTE to deal with category imbalance before using LSTM for churn prediction, and the experimental results showed that SMOTE substantially improved the prediction performance. Aditsania et al.^14^ used Adaptive Synthetic Sampling (ADASYN) to process the data and combined it with the BP algorithm for customer churn prediction and achieved good results.

Despite its excellent performance, a single deep-learning model still has limitations. For example, RNN and LSTM are good at capturing long-term dependencies. Still, they may be inadequate in extracting local features, while CNN, although outstanding in extracting local features, has limited effectiveness in dealing with long-term dependencies. Therefore, it is difficult for a single model to handle long-term dependencies and local feature extraction.

#### Hybrid neural network model

Hybrid neural network models have emerged to overcome the limitations of traditional machine learning and single deep learning algorithms. Such models significantly improve the accuracy and efficiency of churn prediction by combining different types of neural networks. Hybrid neural networks show great potential in this field.

Zhou et al.^19^ proposed a hybrid neural network model (LSTM-CNN) that combines LSTM and CNN, which can capture both long-term dependencies and local features, thus significantly improving the accuracy of prediction. Hu et al.^20^ proposed a PRNN model that combines an RNN with an LSTM and incorporates a product operation to enhance the interaction capability between features. Khattak et al.^27^ further innovated based on LSTM-CNN by using BiLSTM instead of LSTM, which improves the understanding of sequence information and enhances the performance of customer churn prediction. Wu^18^ proposed a hybrid neural network model called HNNSAE that combines the entity embedding layer, feature extraction layer, and MLP; it significantly enhances the richness of feature representation and the ability to capture the correlation information between data using Multi-Head Self-Attention, which significantly improves the prediction performance. Wang et al.^28^ introduced the Attention mechanism into the LSTM model to form an LSTM-Attention model, which enables the model to focus more on key information and improves the understanding of sequential data. Experimental results show that LSTM-Attention outperforms a single LSTM model in a customer churn prediction task. Wang et al.^29^ proposed a FCLCNN-LSTM model, which, by combining the fully-connected layers, CNN, and LSTM, captures features from different layers and performs well in the customer churn prediction task.

These studies show that hybrid neural network models have a promising application in customer churn prediction. They can overcome the limitations of a single deep learning model, understand data features more comprehensively, and improve prediction accuracy and efficiency.

### Research motivations

Although existing models have made some progress in improving the performance of customer churn prediction, they still face several key challenges, mainly including the following three points:

- Class imbalance problem: In actual customer churn datasets, the ratio of churned customers to non-churned customers is usually severely imbalanced. This imbalance can cause the model to overfit the majority class (non-churned customers) and under-identify the minority class (churned customers) during training, thus affecting the overall prediction performance.
- Limitations of feature extraction: existing hybrid neural network models are relatively homogeneous in their customer feature extraction methods, making it difficult to adequately capture the diversity and complexity of customer features and limiting the model’s prediction accuracy.
- Insufficient generalization capability: most of the hybrid neural network studies have been validated only on datasets from a single industry, and the lack of evaluation of generalization performance on datasets from different domains limits the wide application of the model and its practical value.

### Research contributions

To address the above challenges, this study proposes a hybrid neural network-based customer churn prediction model, Customer Churn Prediction Networks (CCP-Net), with the following key contributions:

- Solving the category imbalance problem: In the data preprocessing stage, this study adopts the ADASYN sampling algorithm to effectively address the category imbalance problem in the dataset. Compared with the traditional oversampling method, the synthetic samples generated by ADASYN are closer to the original data distribution. This avoids the model training bias caused by the inconsistent sample distribution, thus improving the negative impact of category imbalance on the prediction performance.
- Enhance feature extraction capability: By combining Multi-Head Self-Attention, BiLSTM, and CNN, CCP-Net gives full play to the advantages of each model, makes up for the deficiencies in the structural design of the existing hybrid neural networks, and significantly improves the comprehensiveness and accuracy of feature extraction. This multi-network synergistic design is of great significance to improve the accuracy of customer churn prediction.
- Enhanced generalization capability: through experiments on datasets from multiple industries (Telecom, Bank, Insurance, News), it is found that the CCP-Net model is not only suitable for churn prediction of Telecom, but also for churn prediction of Bank, Insurance, and News, and shows good prediction performance. This indicates that CCP-Net has a strong generalization ability and has the potential to be widely applied in different industries.

## Proposed methodology

Customer churn prediction involves complex multimodal data, which requires models that can handle multiple feature types simultaneously. Traditional single deep learning models (CNN or LSTM) each focus on different data features, making it difficult to comprehensively handle this type of complex data. Specifically, CNN excels in capturing spatial features and is particularly suitable for processing image data, but has limited effectiveness in processing time-series data; LSTM excels in capturing long-term dependencies in time-series and can effectively process time-series data, but is relatively deficient in extracting local features. In addition, in the face of complex scenarios such as feature interaction and category imbalance, it is often difficult for a single model to achieve ideal prediction performance.

To solve the above problems, we propose the CCP-Net model. CCP-Net consists of three main modules: Multi-Head Self-Attention, BiLSTM, and CNN. The following is a detailed description of the CCP-Net model.

### Multi-head self-attention

**Fig. 1:**
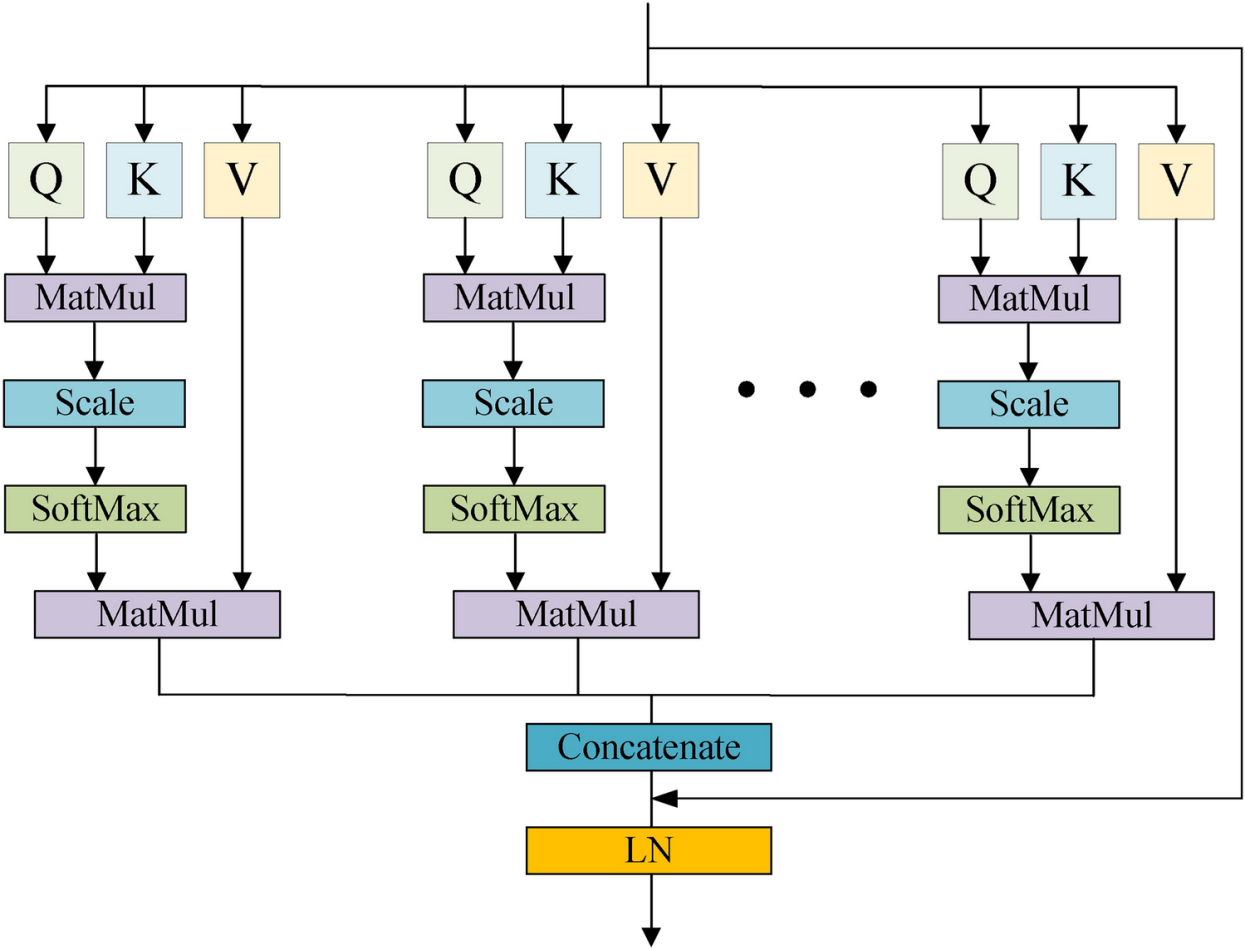
Structure of the improved Multi-Head Self-Attention model.

Since the release of the Transformer model, Multi-Head Self-Attention has received a lot of attention. Multi-Head Self-Attention allows the attention heads to learn in parallel on different subspaces, enabling each attention head to focus on different aspects of the information in the sequence and, through the computation of different weight matrices, to generate finer-grained feature representations that better capture the various dependencies in the sequence. In the task of customer churn prediction, Multi-Head Self-Attention can help the model capture the key features and patterns in customer transaction data more effectively and improve the model’s understanding and prediction of customer churn behavior. The structure of the improved Multi-Head Self-Attention mechanism model is shown in Figure 1:

The computational steps of Multi-Head Self-Attention are as follows:

(1) Projection

The input sequence *X* is linearly mapped to obtain the representation of multiple subspaces respectively.

(2) Attention matrix computation

Attention computation is performed for each projected subspace separately. The attention module of each subspace requires matrix operations, and the output of the attention matrix can be computed using Equation 1, where *Q* denotes the query vector, *K* denotes the key vector,*v* denotes the value vector, and $d_{k}$ denotes the dimension of *K*. The output of each attention head can be derived using Equation 2, where $head_{i}$ denotes the output of the *i*th attention head, and $W_i^QW_i^KW_i^V$ denotes the weight of the *i*th attention head of the *Q*,*K*,*V* vector, respectively.

1 $$\begin{aligned} Attention(Q,K,V)= & Soft\textrm{max}(\frac{QK^T}{\sqrt{d_k}})\cdot V \end{aligned}$$

2 $$\begin{aligned} head_{i}= & Attention(QW_{i}^{Q},KW_{i}^{K},VW_{i}^{V}) \end{aligned}$$

(3) Combination

As shown in Equation 3, the outputs of each attention head are combined by a linear mapping, and *Concat* denotes the combination operation.

3 $$\begin{aligned} MultiHead(Q,K,V)=Concat(head_1,...,head_h) \end{aligned}$$

(4) Output

The final output of Multi-Head Self-Attention is obtained by linear mapping the combined outputs as shown in Equation 4, $W^o$ denotes the weights of the linear mapping.

4 $$\begin{aligned} Output=MultiHead(Q,K,V)\cdot W^{0} \end{aligned}$$

To alleviate the gradient vanishing and promote the information transfer, we introduce the residual connection and Layer Normalization (LN) on Multi-Head Self-Attention, which improves the expressive ability of the model and accelerates the convergence speed, to train the model more effectively. The residual connection and Layer Normalisation are shown in Equation 5 and Equation 6 respectively:

5 $$\begin{aligned} Y= & X+Output \end{aligned}$$

6 $$\begin{aligned} LayerNorm(Y)= & \frac{Y-\mu }{\sigma }\gamma +\beta \end{aligned}$$

$\mu$ and $\sigma$ are the mean and standard deviation of input *Y*, respectively, while $\gamma$ and $\beta$ are learnable parameters.

### BiLSTM

**Fig. 2:**
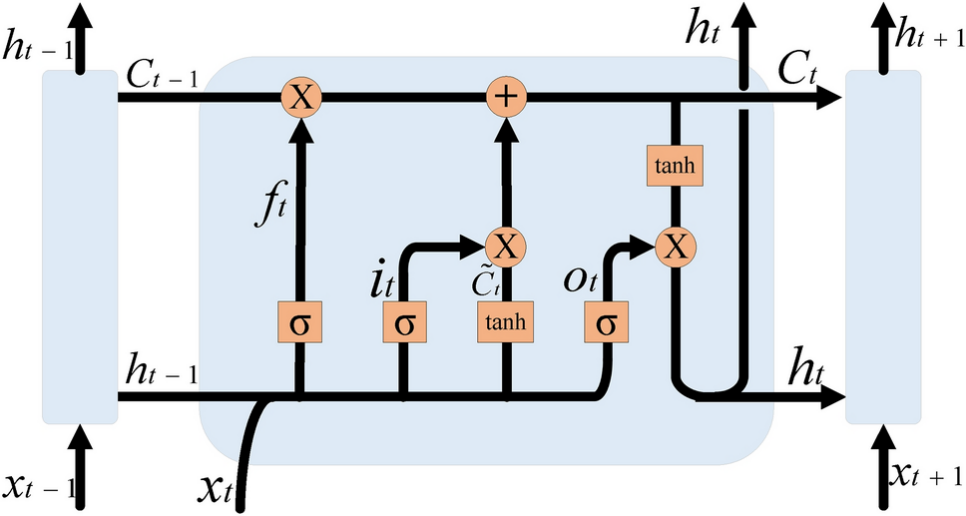
Structure of LSTM model.

BiLSTM processes the input data by forward LSTM and reverse LSTM, in which LSTM has an input gate, forgetting gate, cell gate, and output gate, which can effectively learn and memorize the historical and future temporal information in the data. For example, if a customer has recently made frequent transactions with a company, the probability of churn is significantly reduced; on the contrary, the risk of churn increases. The LSTM model structure is shown in Figure 2:

(1) Forgetting Gate

The function of the forgetting gate is to decide what information to discard from the cell gate. The input of the forgetting gate consists of the output of the hidden state in the previous time step $h_{t-1}$ and the input of the current time step $X_{t}$, which are multiplied with the corresponding weights $W_{fh}$ and $W_{fx}$, respectively, and then added with the bias of the forgetting gate $b_{f}$, which is mapped to the interval [0,1] by the Sigmoid function $\sigma$ to decide the information to be discarded as shown in Equation 7 to get the output of the forgetting gate $f_{t}$.

7 $$\begin{aligned} f_t=\sigma (W_{fh}h_{t-1}+W_{fx}X_t+b_f) \end{aligned}$$

(2) Input gate

The function of the input gate is to determine the information to be stored in the cell gate. The output of the previous time step $h_{t-1}$ and the input of the current time step $X_{t}$ will be multiplied by the corresponding weights $W_{ix}$ and $W_{ih}$, respectively, and then added to the input gate bias $b_{i}$. These values are mapped to the [0,1] interval by the Sigmoid function $\sigma$ indicating the degree of updating the cell gate as shown in Equation 8 to obtain the output of the input gate $i_{t}$.

8 $$\begin{aligned} i_t=\sigma (W_{ix}h_{t-1}+W_{ih}X_{t}+b_{i}) \end{aligned}$$

(3) Cell gate

The cell gate consists of two key steps: computing the new candidate value vector and updating the cell gate.

To compute the new candidate value vector, it is necessary to multiply the hidden state of the previous time step $h_{t-1}$ and the input of the current time step $X_{t}$ with the corresponding weights $W_{ch}$ and $W_{cx}$, respectively, and then add the bias of the cell gate candidate values $b_{c}$, which are mapped by the hyperbolic tangent function *tanh* into the interval [-1, 1] as shown in Equation 9, to generate a new candidate value vector $\tilde{C}_{t}$. This new candidate value vector represents the candidate values to be updated to the cell gate.

9 $$\begin{aligned} \tilde{C}_{t}=\textrm{tanh}(W_{Ch}h_{t-1}+W_{cx}X_{t}+b_{c}) \end{aligned}$$

For the step of updating the cell gate, the cell gate $C_{t-1}$ from the previous time step is multiplied element-by-element with the forget gate $f_{t}$ via $\bigodot$, discarding the information that needs to be discarded. Then the new candidate value $\tilde{C}_{t}$ is multiplied element by element with the input gate $i_{t}$ via $\bigodot$ to add the information that needs to be input. As shown in Equation 10, the two are added together to get the updated cell gate $C_{t}$.

10 $$\begin{aligned} C_t=f_t\odot C_{t-1}+i_t\odot \tilde{C}_t \end{aligned}$$

(4) Output gate

The output gate controls the extent to which the hidden state of the current time step is output to the external output. As shown in Equation 11, the output gate $O_{t}$ is computed from the output of the previous time step $h_{t-1}$ and the input of the current time step $X_{t}$, which are multiplied with the corresponding weights $W_{oh}$ and $W_{ox}$, respectively, and then added to the bias of the output gate $b_{o}$. These values are mapped to the interval [0,1] by the Sigmoid function $\sigma$ indicating which parts of the hidden state $h_{t}$ of the current time step will be output to the outside of the network.

11 $$\begin{aligned} O_{t}=\sigma (W_{oh}h_{t-1}+W_{ox}X_{t}+b_{o}) \end{aligned}$$

As shown in Equation 12, the hidden state $h_{t}$ update is accomplished by the element-by-element product $\bigodot$ and the hyperbolic tangent function *tanh*. The output gate $O_{t}$ controls the extent to which *tanh* acts on the cell gate $C_{t}$ to obtain the hidden state $h_{t}$ for the current time step.

12 $$\begin{aligned} h_t=O_t\odot \tanh (C_t) \end{aligned}$$

**Fig. 3:**
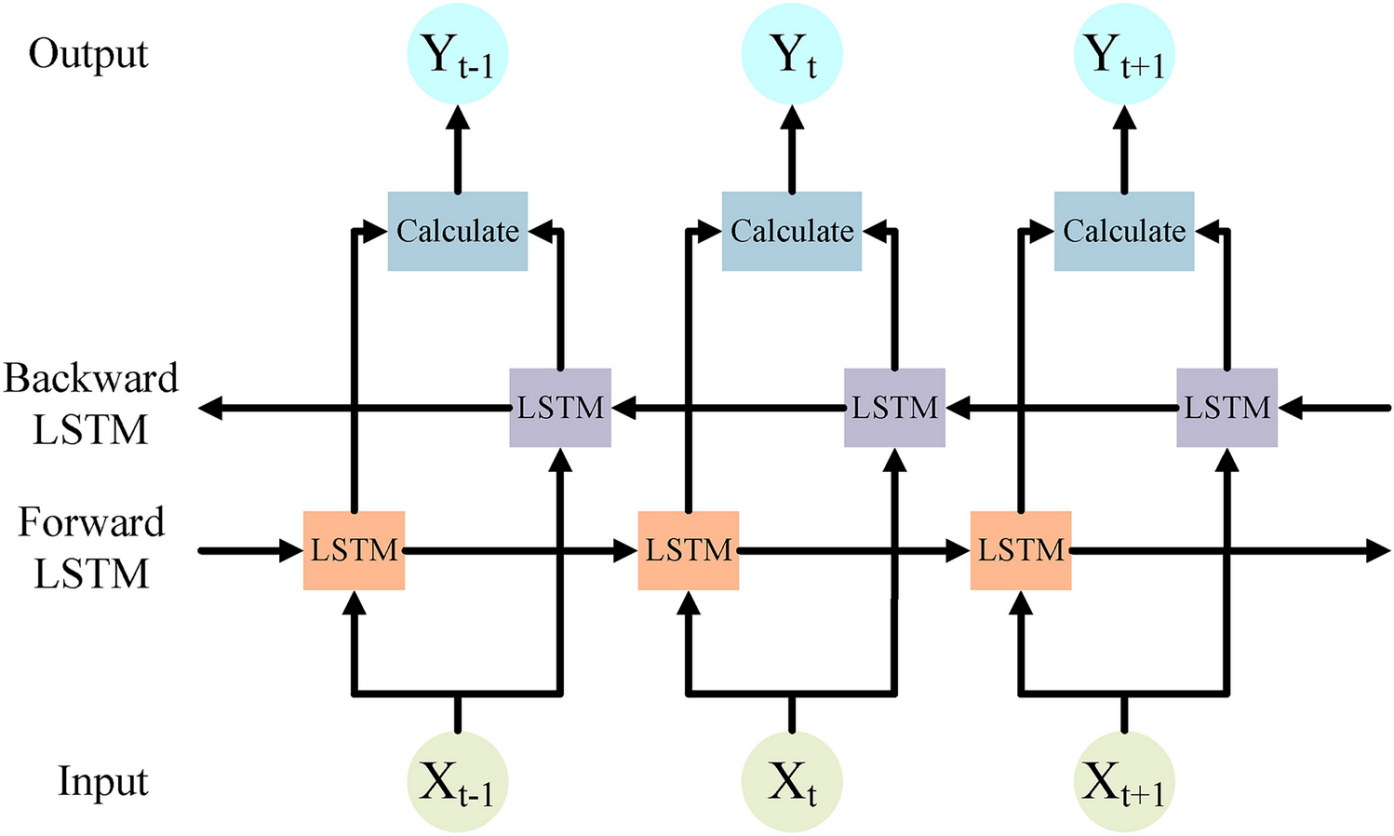
Structure of BiLSTM model.

The BiLSTM model structure is shown in Figure 3.

The output of BiLSTM $Output_{BiLSTM}$ is a concatenation of the hidden state $h_{forward}$ of the forward propagation LSTM and the hidden state $h_{backward}$ of the backward propagation LSTM as shown in Equation 13:

13 $$\begin{aligned} Output_{BiLSTM}=[h_{forward},h_{backward}] \end{aligned}$$

### CNN

**Fig. 4:**
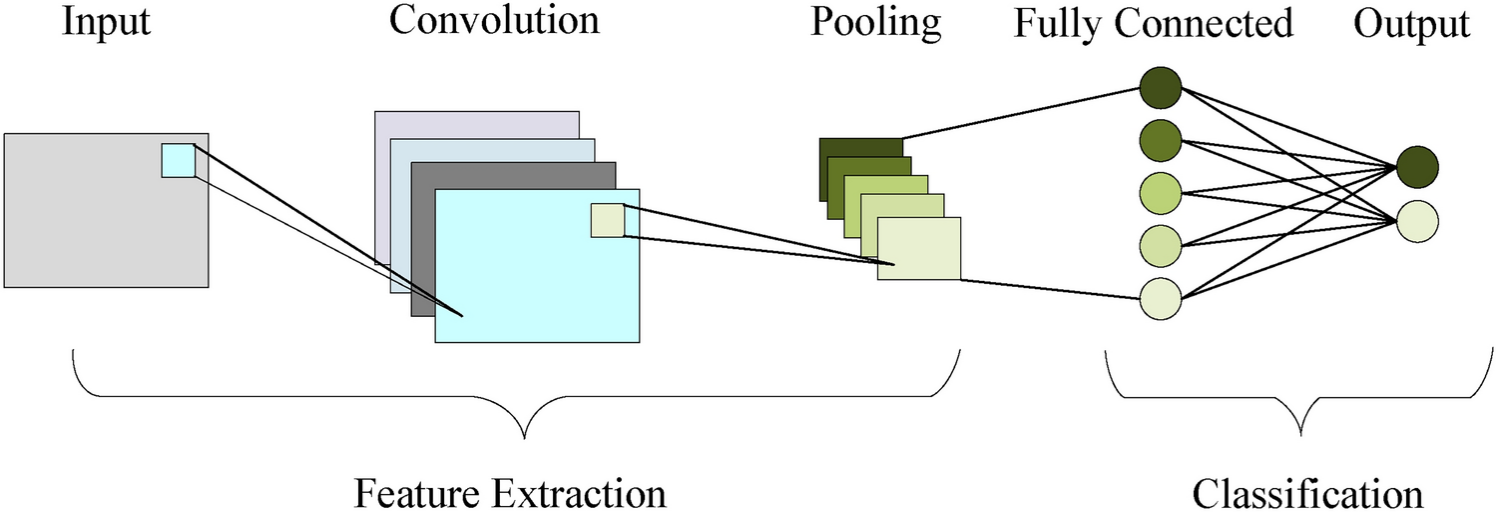
Structure of CNN model.

CNN is a feed-forward neural network, which consists of a convolutional layer, activation layer, pooling layer, and fully connected layer, and can extract the local features of the data through different sizes of filters, effectively learning the spatial structure of the input data and convert it into high-dimensional feature representations, and help the CCP-Net model to learn more complex and advanced features from the transaction data through multiple convolution, activation, and pooling operations. The CNN model structure is shown in Figure 4.

(1) Convolutional layer

The data is fed into the convolutional layer and a convolution operation will be performed to capture the local features and generate the feature map, the convolution operation can be expressed by Equation 14. Where *X* is the input data, *W* is the convolution kernel, the step size is *s*, $Conv_{i}$ is the *i*th neuron in the output of the convolution layer, $X_{i\cdot s+j}$ is the $i\cdot s+j$th element of the input data, $W_{j}$ is the *j*th weight of the convolution kernel and *b* is the bias.

14 $$\begin{aligned} Conv_{i}=\sum _{j=1}^{k}X_{i\cdot s+j}\cdot W_{j}+b \end{aligned}$$

(2) Activation layer

After the convolutional layer, an activation function will be used to introduce nonlinear features to enhance the expressive ability of the neural network, this model uses the *Relu* activation function, which can overcome the problem of gradient vanishing and make the model have a faster training speed, the *Relu* activation function is shown in Equation 15. $A_{i}$ denotes the *i*th neuron in the output of the activation layer;

15 $$\begin{aligned} A_{i}=max(0,Con\nu _{i}) \end{aligned}$$

(3) Pooling layer

The data is fed into a pooling layer that reduces the size of the feature map and the number of parameters while retaining key information; this model uses maximum pooling as shown in Equation 16. *P* is the size of the pooling window, and $P_{i}$ denotes the *i*th neuron output from the pooling layer;

16 $$\begin{aligned} P_{i}=max(A_{i\cdot p:(i+1)\cdot p}) \end{aligned}$$

(4) Fully connected layer

The fully connected layer spreads the previously extracted features to get the prediction result *fc*. As shown in Equation 17. $W_{fc}$ is the weight of the fully connected layer and $b_{fc}$ is the bias of the fully connected layer.

17 $$\begin{aligned} fc=P\cdot W_{fc}+b_{fc} \end{aligned}$$

### CCP-Net model

Existing hybrid neural network models have some shortcomings in their structural design. For example, the PRNN model mainly relies on RNN and LSTM, but RNN and LSTM are prone to gradient vanishing or gradient explosion problems when dealing with long-term dependencies, which limits their performance in customer churn prediction tasks. The HNNSAE model relies only on multi-head self-attention for feature extraction, and the single LSTM-Attention and GRU-Attention models are excellent at time series feature extraction, but are deficient in local feature extraction, resulting in limited prediction accuracy. In contrast, the LSTM-CNN, BiLSTM-CNN, and FCLCNN-LSTM models are capable of extracting both local and global features, but are still deficient in dealing with complex global dependencies, while Multi-Head Self-Attention is more advantageous in capturing complex global dependencies, which enhances the model’s feature extraction capability.

**Fig. 5:**
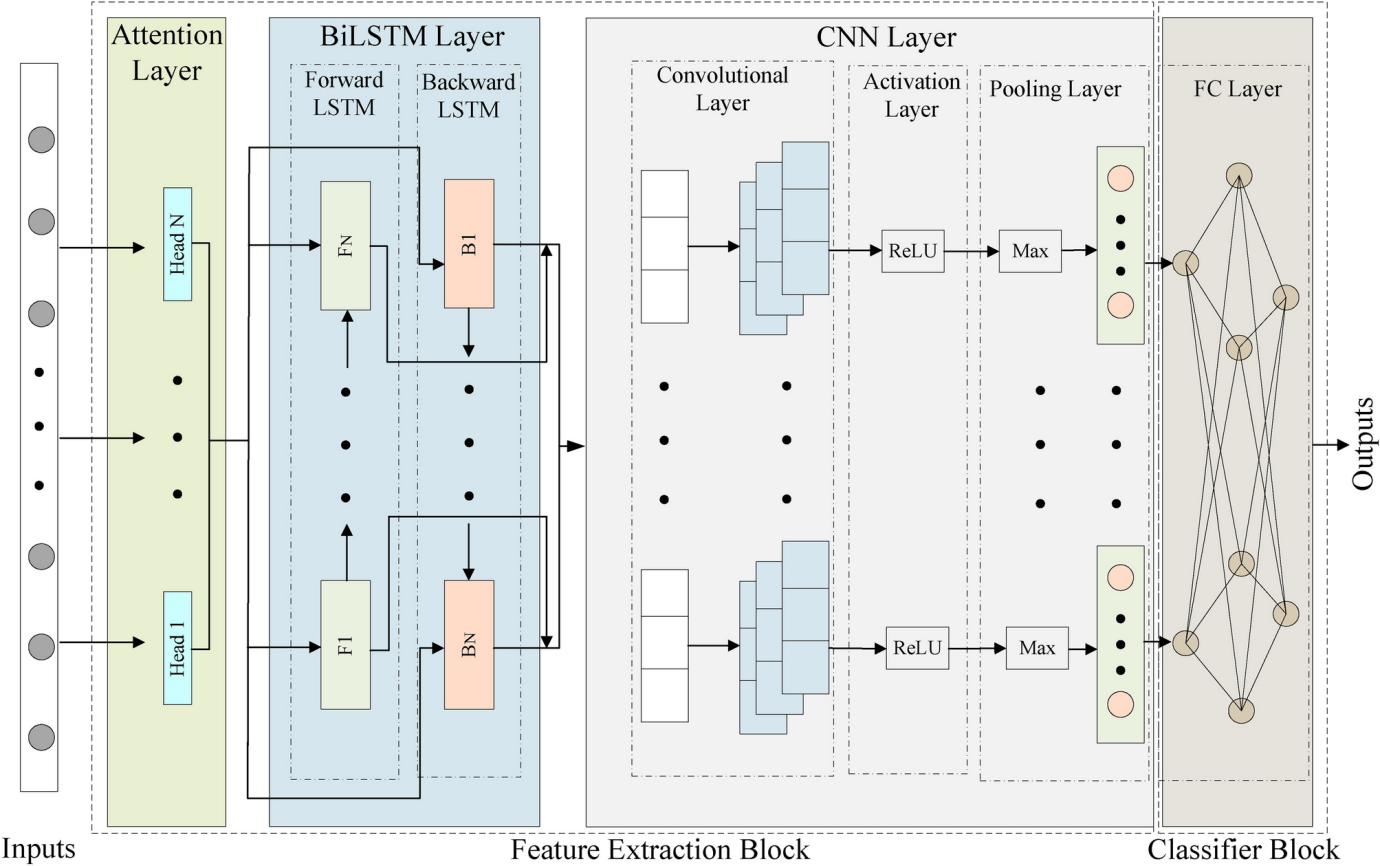
Structure of CCP-Net model.

To address these limitations, this study proposes a customer churn prediction network model called CCP-Net, CCP-Net combines three types of neural networks: Multi-Head Self-Attention, BiLSTM, and CNN. The structure of CCP-Net is shown in Figure 5.

- Multi-Head Self-Attention Module:
  CCP-Net first uses the Multi-Head Self-Attention module to process customer transaction sequences. This module focuses on different parts of the information in the sequence by decomposing the input sequence into multiple attention heads that learn in parallel in different subspaces. Compared with the single-attention mechanism, Multi-Head Attention can capture the complex relationships between features and effectively extract key information and potential patterns in the transaction data by weighted summation of the outputs of individual attention heads.
  Multi-Head Self-Attention can capture global dependencies and process information in parallel, avoiding the information decay triggered by the step-by-step processing of traditional sequential models (e.g., LSTM and GRU). In addition, the multi-head mechanism enables the model to focus on the contextual information of different parts of the input sequence to fully capture the complex dependencies between features, especially in the task of customer churn prediction, which helps to capture global contextual information in long time series, such as customers’ historical behaviors, consumption habits, and long-term dependencies. In this way, the model can capture complex dependencies quickly, avoiding the problem of gradient vanishing or gradient explosion, resulting in a more comprehensive understanding of customer behavior patterns.
- BiLSTM module:
  The output of the Multi-Head Self-Attention is then passed to the BiLSTM module, which, by processing the input sequences in both directions, can take into account both the information flows from the past and the future, leading to a more comprehensive understanding of the context of the sequence data. Compared to BiGRU, BiLSTM introduces three important gating mechanisms - input gates, forgetting gates, and output gates - which effectively manage the storing and forgetting of information to ensure that important contextual information is retained over long sequences. This gives BiLSTM a significant advantage in dealing with complex long-term dependencies, especially in customer churn prediction, where customer churn behavior is usually influenced by multiple factors that may be distributed at different locations in the time series.
  Through the two-way propagation mechanism, BiLSTM is not only able to capture past behavioral information but also predict future changes, which comprehensively improves the model’s prediction capability. In contrast, although BiGRU is also capable of bi-directional processing, it has a relatively simplified structure and relies only on update gates and reset gates to control the flow of information, which reduces the amount of computation, but BiGRU is more limited than BiLSTM in the modeling of long-term dependencies. As a result, BiLSTM is more suitable for capturing long-term behavioral patterns in customer churn prediction and can more accurately model important features in long-term time series.
- CNN module:
  The output of the BiLSTM module is then passed to the CNN module, which extracts local features through convolutional operations to capture short-term patterns in the transactional data. The CNN’s translation invariance enables it to identify local features efficiently. It combines with the ReLU activation function to introduce nonlinearities and reduce the feature dimensions through a maximal pooling operation to transform the local features into a higher-dimensional representation. Through multi-layer convolution operation, CNN performs layer-by-layer feature extraction on the input data and can discover important local patterns. In the task of churn prediction, local features such as short-term behavioral patterns and immediate changes in customer interactions are also critical, and CNNs help the model better understand short-term customer behavioral changes by capturing these local features, which enhances the model’s prediction capability.

In summary, by combining the advantages of Multi-Head Self-Attention, BiLSTM, and CNN, CCP-Net overcomes the deficiencies of existing models in capturing global and local dependencies and comprehensively improves the performance of customer churn prediction. Multi-Head Self-Attention provides a global perspective, helping the model to capture complex customer behavior patterns; BiLSTM strengthens long-term dependency learning in time series and effectively handles multi-dimensional information of customer churn; CNN excels in local feature extraction, enabling the model to achieve efficient learning at different feature levels. Through this comprehensive design, CCP-Net not only significantly improves the prediction accuracy, but also enhances its applicability in practical applications.

## Experiment

**Fig. 6:**
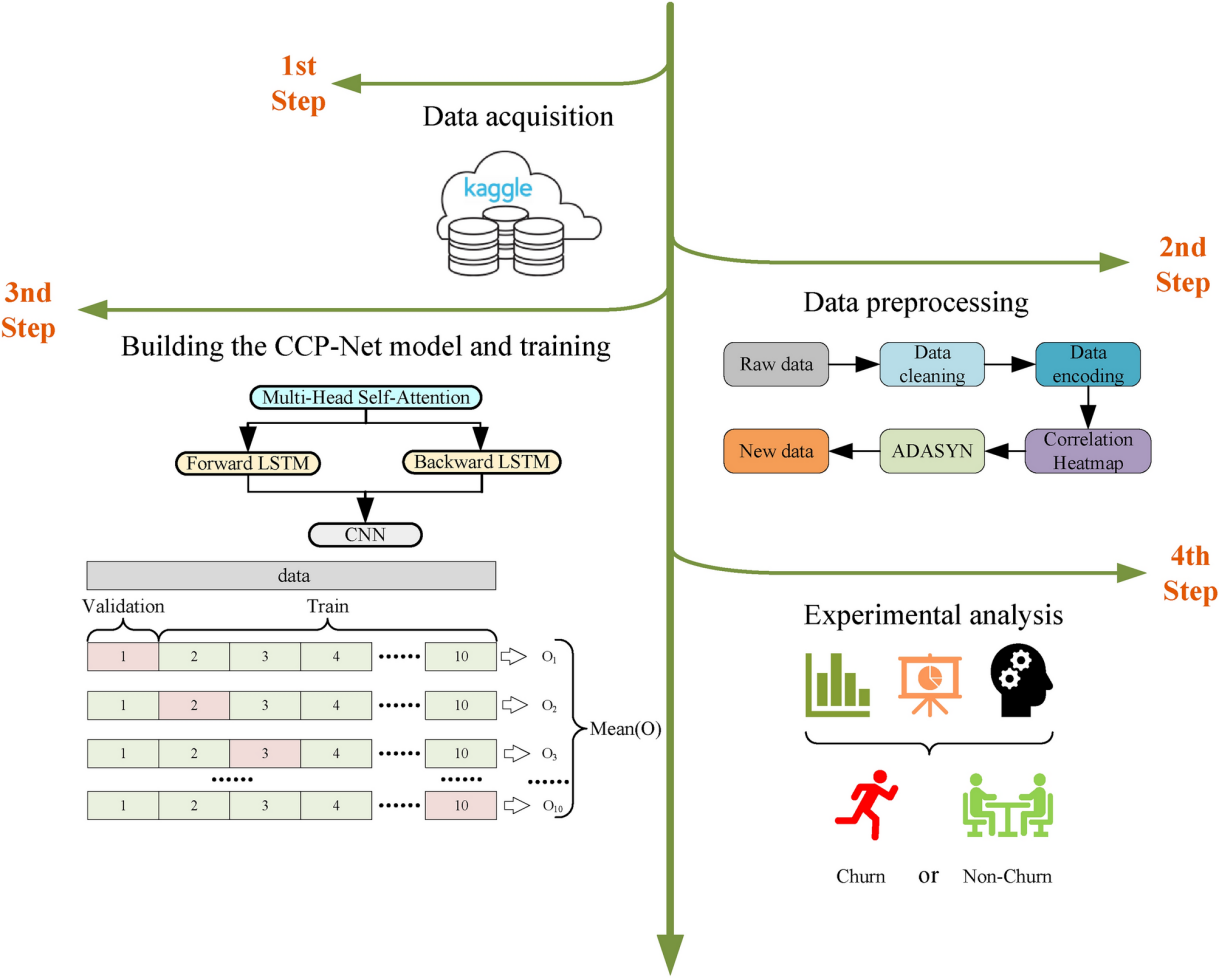
Flow chart of the experiment.

The experiment is divided into the following four steps:(1) Data acquisition, (2) Data preprocessing, (3) Building the CCP-Net model and training, and (4) Experimental analysis. The flow of the experiment is shown in Figure 6.

### Data acquisition

Four customer churn datasets from the Kaggle data science competition platform are used in this study, including Telecom^30^, Bank^31^, Insurance^32^, and News^33^. These datasets are widely used in the field of customer churn prediction, facilitating the comparison of the model proposed in this paper with other models. Table 1 summarises the details of each dataset and shows that all these datasets suffer from significant category imbalance.

- The Telecom dataset contains customers’ transaction frequency, service usage, and churn history.
- The Bank dataset records customers’ transaction behavior, account balances, and other financial activities.
- The Insurance dataset provides historical information about customers and product usage.
- The News dataset includes information about users’ reading habits, language, address, and more.

### Data preprocessing

Data preprocessing is a critical step to ensure model performance. Our preprocessing steps for the four datasets are as follows:

**Table 1:** Dataset information.

Dataset	sample size	number of features	number of churned customers	number of non-churned customers	churn rate
Telecom	7043	21	1869	5163	26.58%
Bank	10000	14	2037	7963	20.37%
Insurance	33908	17	3967	29941	11.70%
News	15855	19	3037	12818	19.15%

#### Data cleansing

In our initial data exploration, we found a small number of missing and duplicate values in the dataset. Since these problematic data accounted for a relatively small percentage of the data, it was straightforward to remove rows that contained missing or duplicate values. For example, the ’Language’, ’weekly fee’, and ’Nielsen Prizm’ columns in the News dataset had some missing values, and deleting the corresponding rows was sufficient. In addition, entries with similar but different expressions are unified, e.g., in the Telecom dataset, ’No internet service’ and ’No phone service’ are unified as ’No’.

#### Data encoding

The dataset contains both numeric and categorical data.

- For numerical data, we used standardization to adjust their mean to 0 and variance to 1 to ensure the consistency of different features on the numerical scale.
- For category-based data, since the format of category-based data is string-based, it cannot be directly used for further calculations, so we need to convert the string data to numeric representation through label encoding and convert the string format of category-based data to numeric representation so that the model can recognize the relationship between different categories. For example, in the Bank dataset, the category values of ’Geography’ and ’Gender’ are encoded as 0, 1, 2, etc.

#### Feature selection

To reduce feature dimensionality and remove noise, we use correlation heatmaps to identify features with low correlation with the target variable. Heatmap is a visualization tool that demonstrates the correlation between features through shades of colors, and its core principle is based on the calculation of the correlation coefficient, which is used to measure the strength and direction of the linear relationship between two variables and takes the value in the range of [-1, 1]. Specifically, 1 indicates a perfect positive correlation, -1 indicates a perfect negative correlation, and 0 indicates no correlation.

By analyzing the correlation coefficients on the correlation heatmap, we can exclude feature columns with low correlation with the target variable, thus avoiding overfitting the model to unimportant features. This strategy not only reduces the training time but also enhances the generalization ability of the model. The main reason for choosing to remove features with correlation coefficients below a specific threshold is to reduce noise and improve the validity and interpretability of the model. Features with low correlation may not have a significant impact on the prediction of the target variable, and retaining them may introduce interference and lead to a decrease in model performance. By removing unimportant features, the model can focus more on features that are more relevant to the target variable, thus improving generalization on new data and reducing the risk of overfitting, as well as shortening training time.

**Fig. 7:**
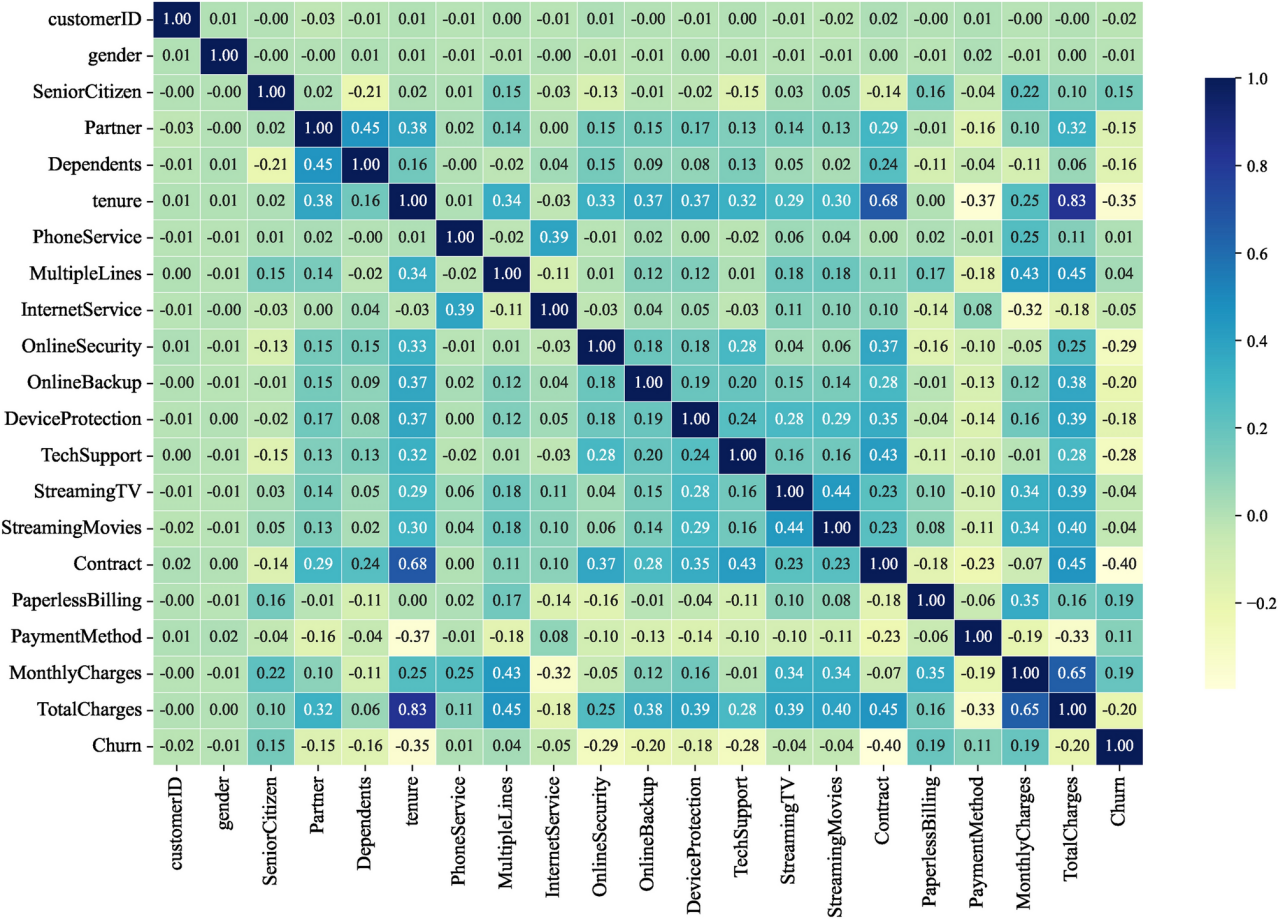
Heatmap of Telecom dataset correlation.

**Fig. 8:**
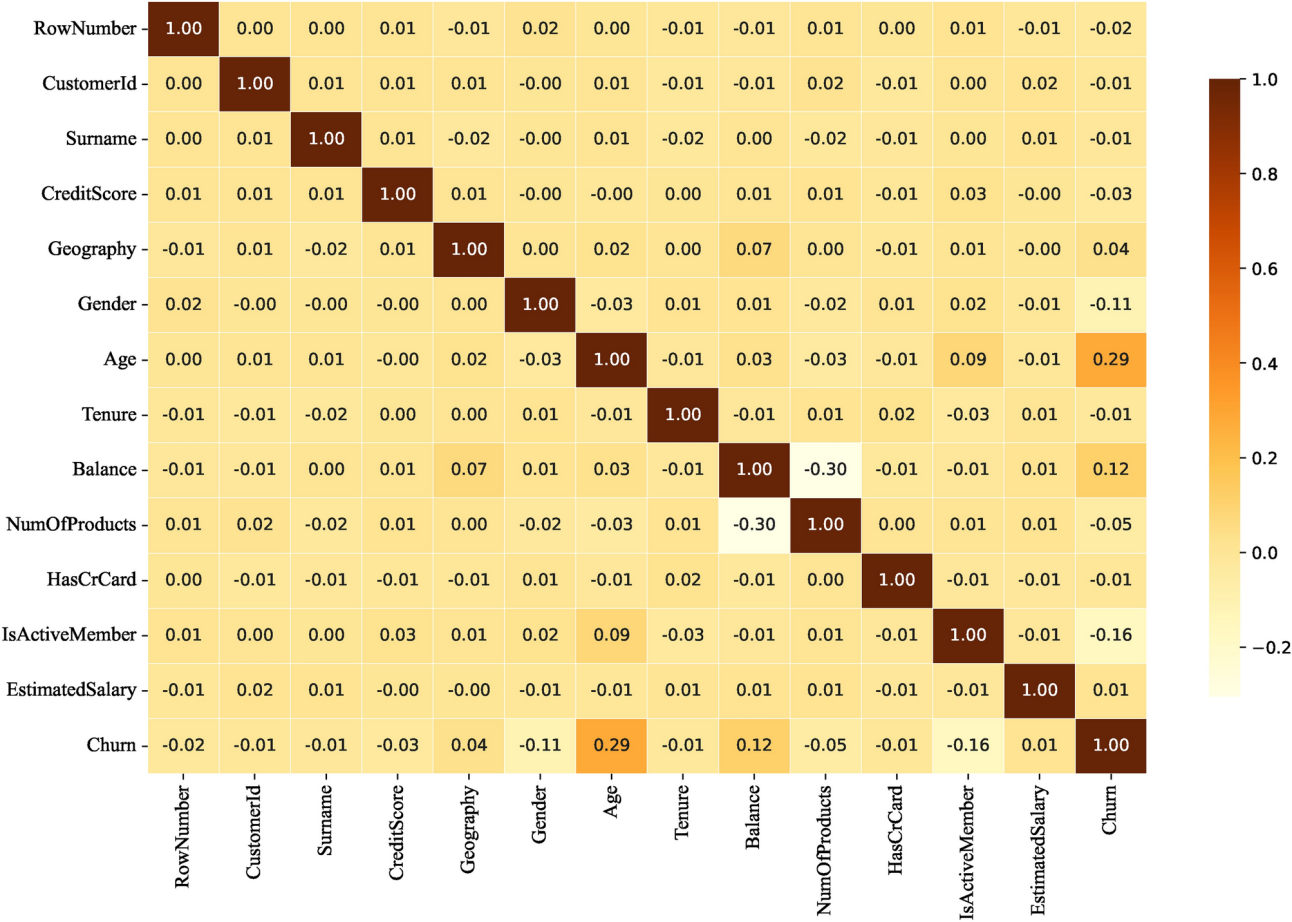
Heatmap of Bank dataset correlation.

**Fig. 9:**
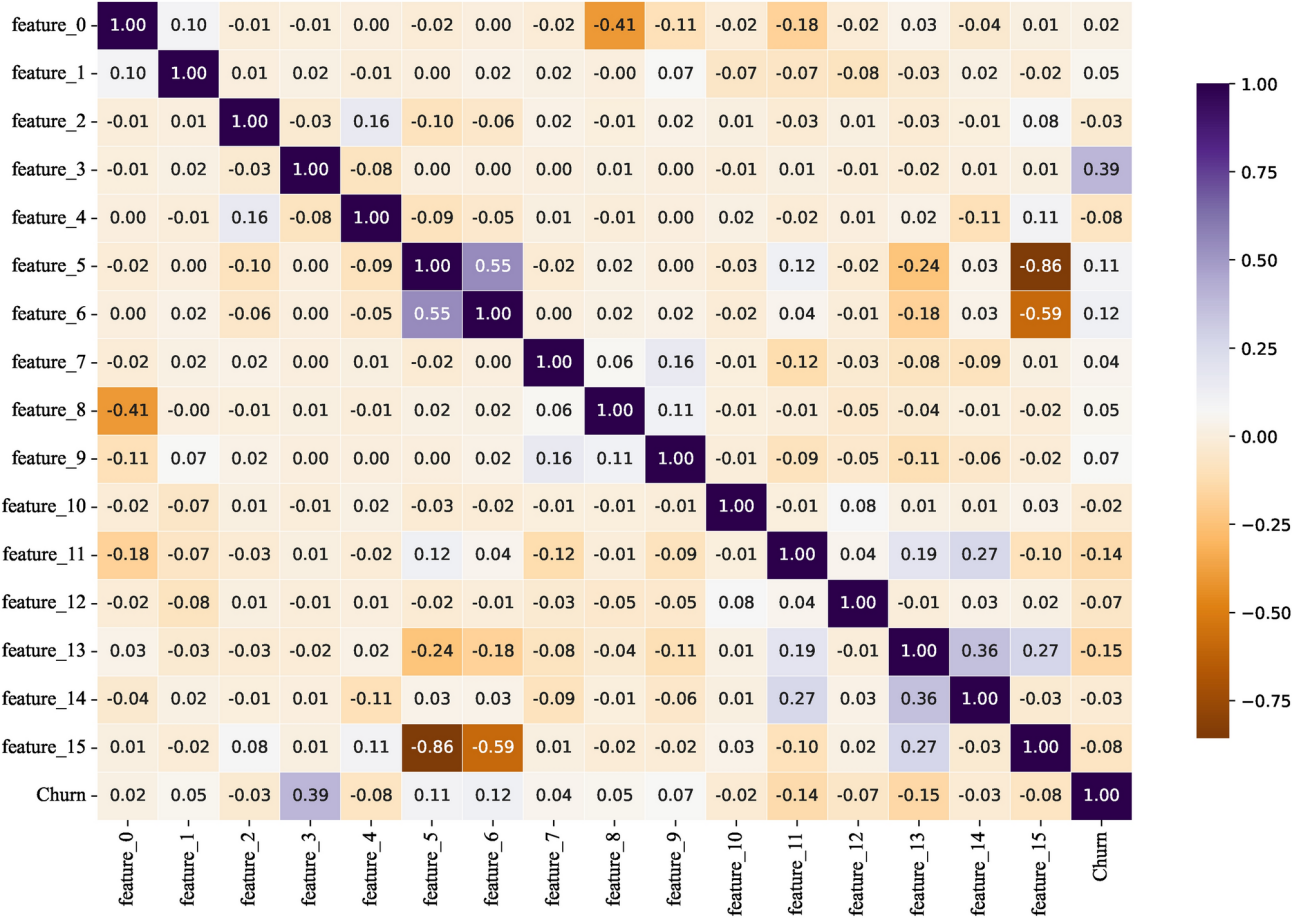
Heatmap of Insurance dataset correlation.

**Fig. 10:**
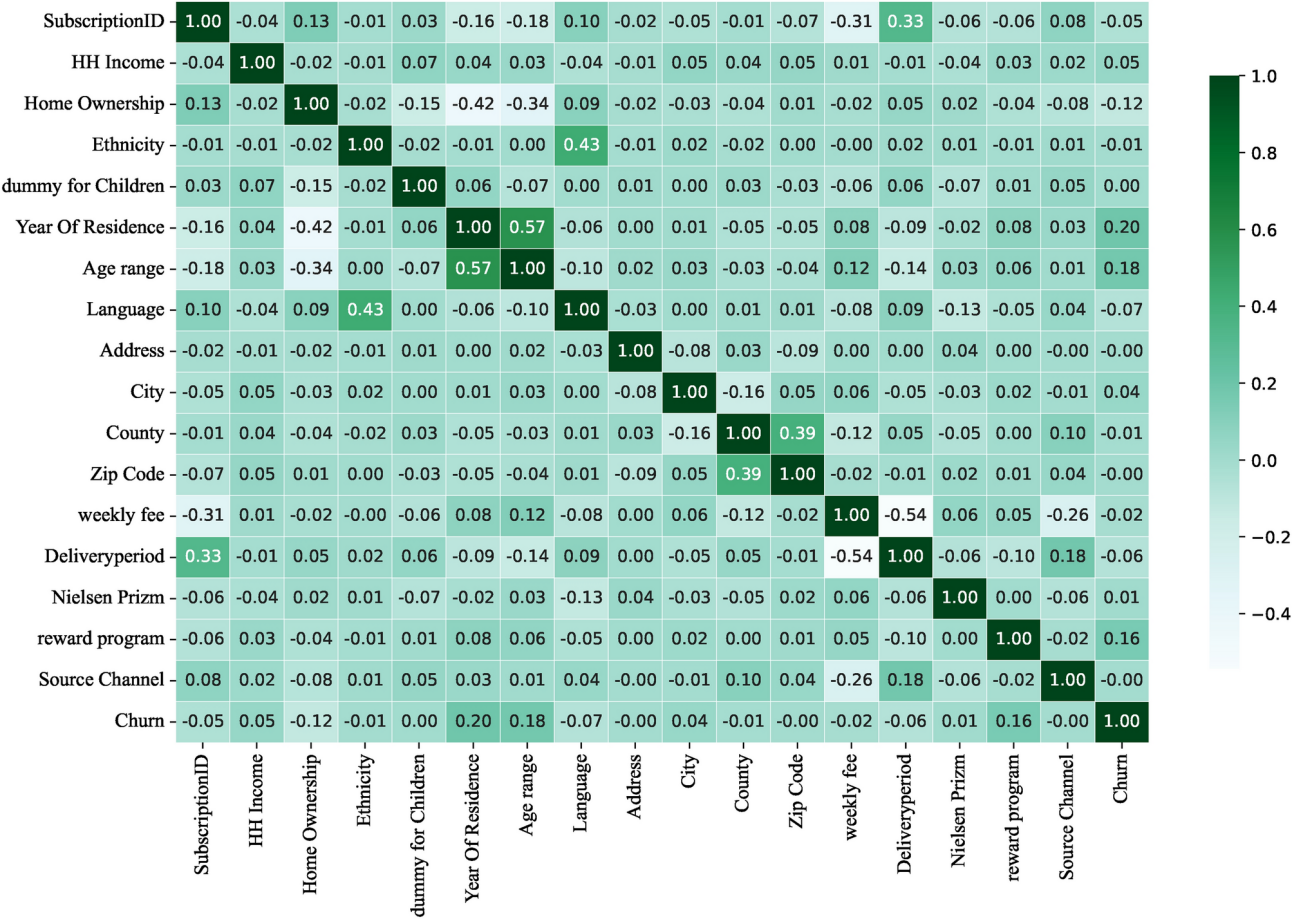
Heatmap of News dataset correlation.

Figures 7, 8, 9, 10 show the correlation heatmaps for the datasets Telecom, Bank, Insurance, and News, respectively.

- Telecom dataset
  In the Telecom dataset, we removed feature columns with correlation coefficients less than 0.1, including ‘customerID’, ‘Gender’, ‘PhoneService’, ‘MultipleLines’, ‘InternetService’, ‘StreamingTV’, and ‘ StreamingMovies’. These features are not considered important for the following reasons:
  ‘customerID’: this feature is a unique identifier for each customer and does not provide meaningful correlation information when predicting churn. It is only an identifier and does not reflect the customer’s behavior or other characteristics that can be used for churn prediction.
  ‘Gender’ and ‘PhoneService’: while gender and having a phone service not may have an impact on customer behavior in some scenarios, in this dataset these features are weakly correlated with churn, showing a low correlation that may lead to overfitting of the model or learning to noise.
  ‘MultipleLines’, ‘InternetService’, ‘StreamingTV’, and ‘StreamingMovies’: these features are more indirectly related to whether or not a customer is churning, and the correlation is low, especially when there is no further data to support it (e.g., the customer’s specific usage). For example, whether or not a customer uses a particular service does not always reflect whether or not they will churn, and therefore these features were not considered sufficiently important in this experiment.
- Bank dataset
  For the Bank dataset, we removed feature columns with correlation coefficients less than 0.03: ‘CustomerId’, ‘Surname’, ‘Tenure’, ‘HasCrCard’, and ‘EstimatedSalary’. The reason for deleting these features is as follows:
  ‘CustomerId’ and ‘Surname’: these two features are unique identifiers or surnames of customers and are unsuitable for predicting churn as they do not contain any key information about customer behavior or correlation to churn. As identifying information only, they have no real value for model training.
  ‘Tenure’ and ‘EstimatedSalary’: the low correlation of these features suggests that they contribute less to churn prediction.’ Tenure’ may be correlated with customer churn, but the correlation with churn is weak in this dataset. ‘EstimatedSalary’ also has limited predictive power and may not be as meaningful as other financial or behavioral characteristics of customers.
- Insurance dataset
  In the Insurance dataset, we removed feature columns with correlation coefficients less than 0.05: “feature_0”, “feature_2”, “feature_7”, “feature_10”, and “feature_14”. The reasons for the deletion of these features are as follows:
  “feature_0”, “feature_2”, “feature_7”, “feature_10”, and “feature_14”: these features had a weak correlation with the target variable (churn) in the preliminary analysis. They do not provide enough information to help distinguish whether customers are churning or not, and may simply be noise or irrelevant information in the data. Retaining these features introduces unnecessary complexity and leads to a reduction in model performance, so they were chosen to be removed.
- News dataset
  For the News dataset, we removed feature columns with correlation coefficients less than 0.05: ‘Ethnicity’, ‘dummy for Children’, ‘Address’, ‘City’, ‘County’, ‘Zip Code’, ‘weekly fee’, ‘Nielsen Prizm’ and ‘ Source Channel’. The reasons for removing these features are as follows:
  ‘Ethnicity’ and ‘dummy for Children’: these two features are of low relevance and may introduce bias or sensitivity issues, particularly in social science data. They fail to provide a meaningful contribution to the prediction of attrition rates.
  ‘Address’, ‘City’, ‘County’, and ‘Zip Code’: this geolocation information is usually not directly related to customer behavior, especially when there are no other details about the customer’s location. Whilst they may be valid in some specific scenarios, in the current dataset they are less relevant and tend to introduce noise.
  ‘weekly fee’, ‘Nielsen Prizm’, and ‘Source Channel’: these features are weakly correlated and may not be directly related to the target variable (churn). In particular, ‘weekly fee’ and ‘Nielsen Prizm’ are not very useful in predicting churn and may simply reflect non-critical user behavior information.

Through this series of feature selection processes, we not only improve the training efficiency of the model but also enhance its generalization ability. Removing irrelevant or low-relevance features makes the model more focused on features that are more relevant to the target variable, which ultimately improves the accuracy of the prediction and reduces the risk of overfitting.

#### Data balancing

In the four datasets (Telecom, Bank, Insurance, and News) used in the experiment, the churn rates are 26. 58%, 20. 37%, 11. 70% and 19. 15%, respectively, all of which suffer from a significant category imbalance. To address this issue, this experiment uses the ADASYN technique to balance the dataset.

ADASYN is an improved oversampling technique, the core idea of which is to generate new synthetic samples by interpolating the feature space of a small number of class samples. Compared with the traditional SMOTE algorithm, ADASYN introduces a dynamic consideration mechanism of sample importance, which adjusts the number of synthetic samples generated according to the importance of each minority class sample in the classification. This dynamic adjustment mechanism makes the model pay more attention to those samples that have a critical impact on the classification task during the training process, effectively reducing the risk of overfitting to the majority class.

ADASYN pays special attention to the difficulty and criticality of minority samples when generating synthetic samples. Unlike traditional oversampling methods, ADASYN determines the importance of the minority samples based on their neighborhood density, which in turn determines the number of synthetic samples to be generated. Specifically, ADASYN calculates the neighbourhood density $D(x_i)$ for each minority class sample $x_i$,as shown in Equation 18:

18 $$\begin{aligned} D(x_i)=\frac{|N_k(x_i)|}{|N_k(x)|} \end{aligned}$$

$|N_k(x_i)|$ denotes the number of minority class samples adjacent to sample $x_i$, and $|N_k(x)|$ is the total number of neighbors of all minority class samples. Based on this density, ADASYN can generate the number of synthetic samples $N_{new}(x_i)$ for each minority class sample, as shown in Equation 19.

19 $$\begin{aligned} N_{\text {new}}(x_i) = D(x_i) \cdot N_{\text {total}} \end{aligned}$$

This adjustment mechanism allows more synthetic samples to be generated for hard-to-classify samples near the decision boundary, thus helping the model to pay more attention to these ‘boundary’ samples and improve its ability to recognize a small number of classes.

In addition, by generating more synthetic samples for these critical boundary samples, ADASYN enhances the model’s performance in these hard-to-distinguish regions and improves its overall generalization. In this way, ADASYN not only balances the class distribution but also significantly improves the recognition of minority classes.

After determining the number of generated samples, ADASYN selects the closest *k* neighbor samples $\{x_{i_1}, x_{i_2}, \ldots , x_{i_k}\}$ that are closest to each of the minority class samples $x_i$. Then, synthetic samples $x_{new}$ are generated by Equation 20:

20 $$\begin{aligned} x_{\text {new}} = x_i + \lambda (x_j - x_i) \end{aligned}$$

Where $x_i$ is one of the selected neighbors and $\lambda$ is a random number between 0 and 1 indicating that the newly generated sample is located somewhere between $x_i$ and its neighbor $x_j$.

**Fig. 11:**
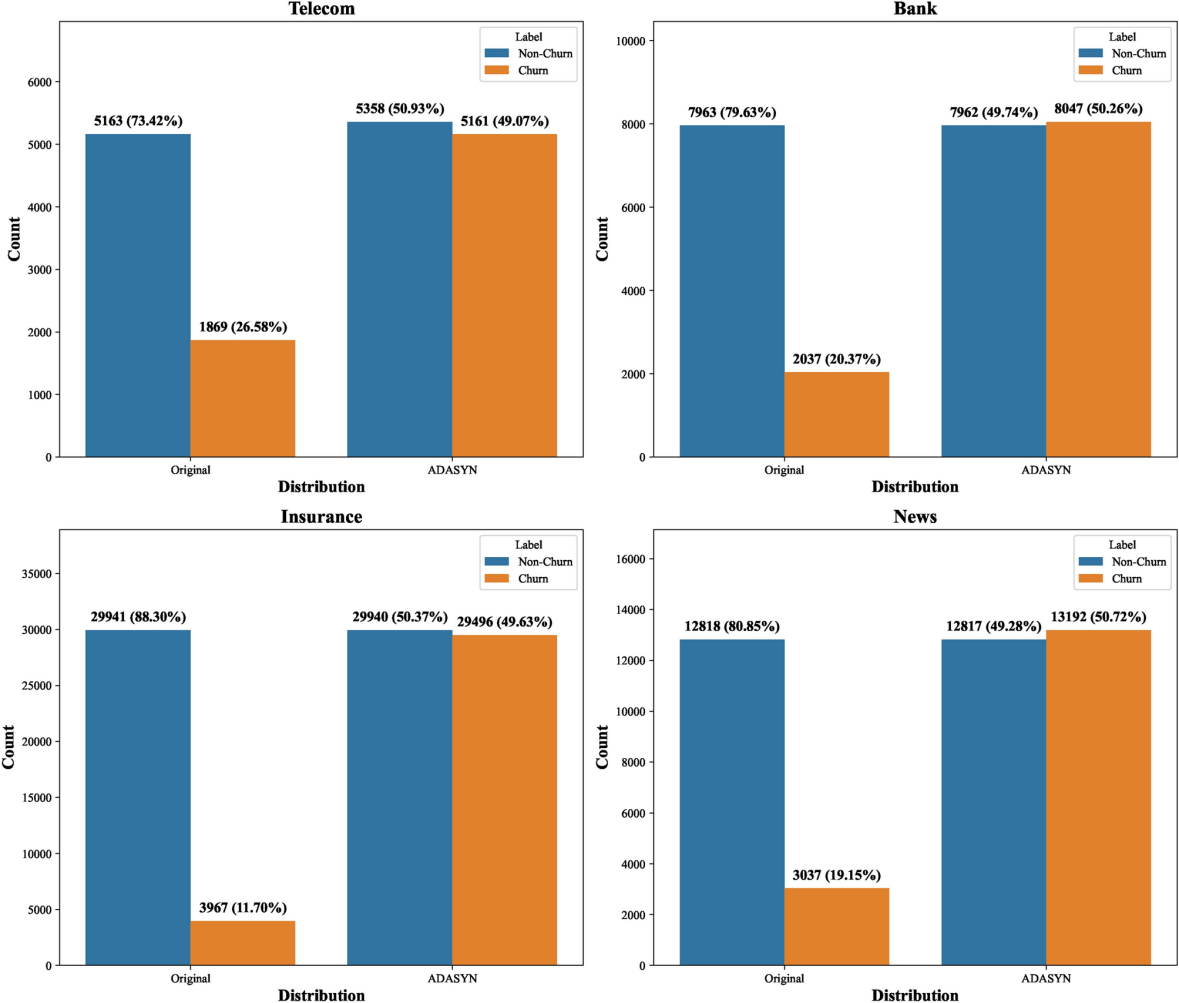
Changes in category distribution before and after treatment with ADASYN.

Figure 11 show the changes in the distribution of categories before and after the application of ADASYN, further validating the effectiveness of ADASYN in balancing the dataset.

### Building the CCP-Net model and training

In this experiment, we choose to use Jupyter Notebook as the development environment and conduct the experiment on the Windows 10 operating system. Data preprocessing, model evaluation, and the implementation of machine learning algorithms are all based on the Scikit-learn library, while the construction and training of the CCP-Net model are based on the PyTorch framework. To ensure the training efficiency and reliability of the results, this experiment is configured with two Intel Xeon E5-2620 v4 processors, 64 GB of RAM, and four NVIDIA Tesla M40 GPUs to accelerate the model training and processing of large-scale datasets.

In the output phase of the CCP-Net model, a *Sigmoid* activation function is used to limit the prediction results between 0 and 1, as shown in Equation 21:

21 $$\begin{aligned} y=Sigmoid(W^T\cdot x+b) \end{aligned}$$

Where if the output value is less than 0.5, it means that the customer will not churn; if the output value is greater than or equal to 0.5, it means that the customer has a high probability of churn. This probability prediction provides a clear indication of the binary classification problem and facilitates subsequent decision-making.

For the task of predicting customer churn in binary classification, we choose Binary Cross Entropy Loss (BCELoss) as the loss function to quantify the difference between the model predictions and the true labels. This loss function is defined as shown in Equation 22:

22 $$\begin{aligned} L=-[y\cdot \log {(\hat{y})}+(1-y)\cdot \log {(1-\hat{y})}] \end{aligned}$$

Where $\hat{y}$ denotes the predicted output of the model,*y* denotes the true labels, and *L* is 0 when *y* is equal to 1, indicating no loss.

To improve the generalization ability of the model, a 10-fold cross-validation method was used in this study. Specifically, the dataset is divided into ten equal parts, and each time, one part is kept as the validation set, and the remaining nine parts are used as the training set. This process is repeated ten times and the average of the ten experimental results is calculated to ensure the stability and reliability of the results.

During the training process, we use Adam optimizer, an optimization algorithm that combines momentum and adaptive learning rate to accelerate model training and improve convergence speed. The initial learning rate is set to 0.001 and is dynamically adjusted during training to maintain model stability. In addition, we introduce the Early Stopping technique to prevent model overfitting. When the validation set loss does not improve significantly within some epochs, the training is stopped early to save computational resources and improve model stability. The pseudo-code of the CCP-Net model is shown in Algorithm 1.

**Algorithm 1:**
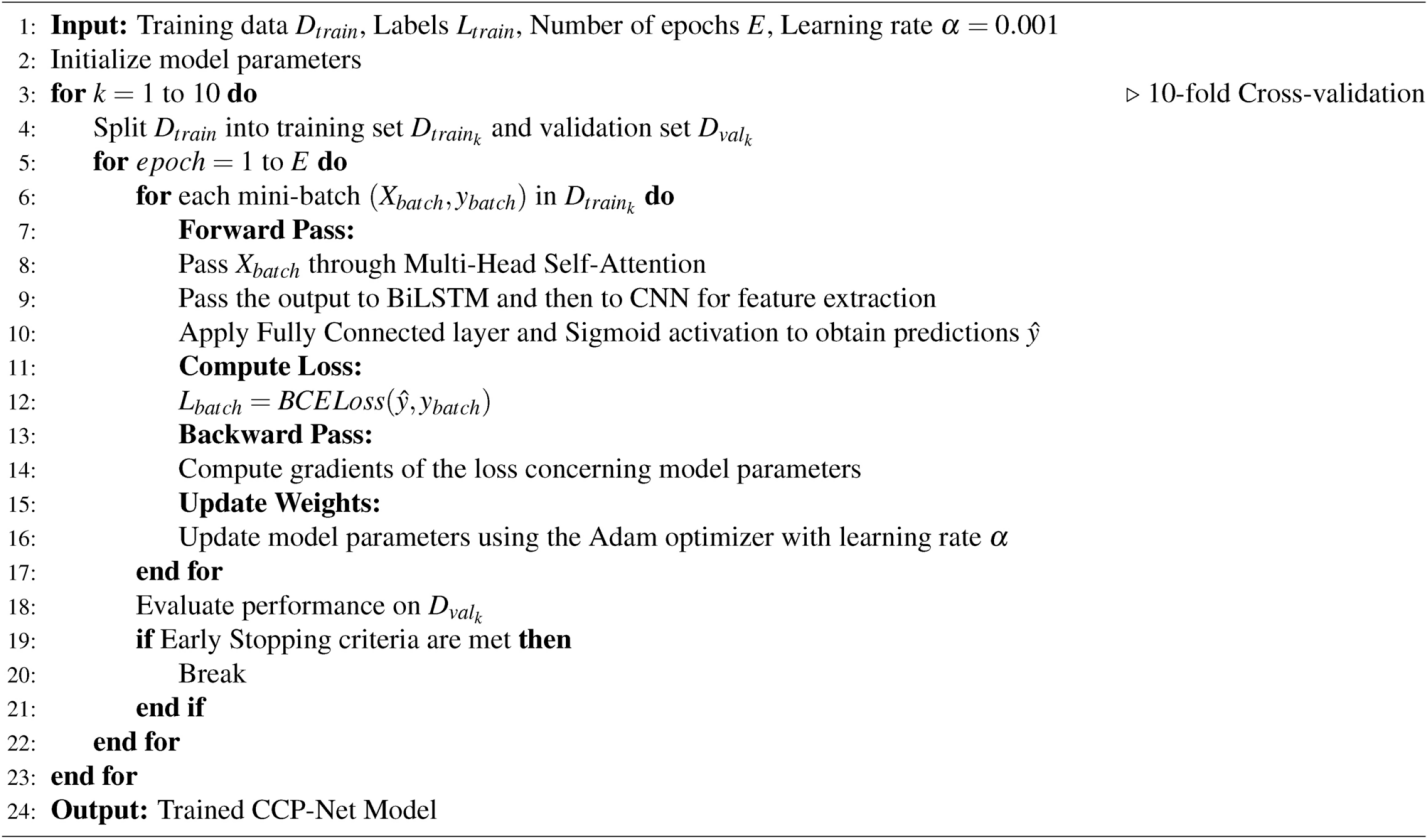
Training Process for CCP-Net Model.

## Experimental analysis

### Evaluation metrics

In the customer churn prediction task, the evaluation of the model performance can be achieved using a confusion matrix, which consists of four key components: True Positives (TP), True Negatives (TN), False Positives (FP), and False Negatives (FN). Among them:

- TP represents the number of samples correctly predicted as churn customers.
- TN represents the number of samples correctly predicted as non-churning customers.
- FP represents the number of samples incorrectly predicted as churn customers.
- FN represents the number of samples incorrectly predicted as non-churning customers.

Table 2 demonstrates the structure of the confusion matrix:

**Table 2:** Confusion matrix.

Real	Prediction	
	Churn	Non-Churn
Churn	TP	FN
Non-Churn	FP	TN

To comprehensively evaluate the performance of different classification algorithms in customer churn prediction, the following four main evaluation metrics are selected in this study: Accuracy, Precision, Recall, and F1. These metrics can reflect the model’s performance in the churn prediction task from multiple perspectives.

- Accuracy refers to the proportion of samples correctly predicted by the model to the total samples, as shown in Equation 23. For the churn prediction task, Accuracy is used to measure the overall prediction accuracy of the model.

23 $$\begin{aligned} Accuracy=\frac{TN+TN}{TP+TN+FP+FN} \end{aligned}$$

- Precision is the proportion of actual churn customers in the sample predicted as churn by the model, as shown in Equation 24. In churn prediction, a higher Precision means that the model has a lower false alarm rate in churn prediction.

24 $$\begin{aligned} Precision=\frac{TP}{TP+FP} \end{aligned}$$

- Recall is the proportion of actual churn customers successfully predicted as churn customers by the model, as shown in Equation 25. In churn prediction, a higher Recall indicates that the model performs well in capturing real churn customers and has a lower underreporting rate.

25 $$\begin{aligned} Recall=\frac{TP}{TP+FN} \end{aligned}$$

- F1 is the reconciled average of Precision and Recall, as shown in Equation 26. F1 integrates the model’s prediction accuracy and ability to capture real churn customers, and can effectively assess the overall performance of the model.

26 $$\begin{aligned} F1=2\times \frac{Precision\times Recall}{Precision+Recall} \end{aligned}$$

### Analysis of experimental parameters

**Fig. 12:**
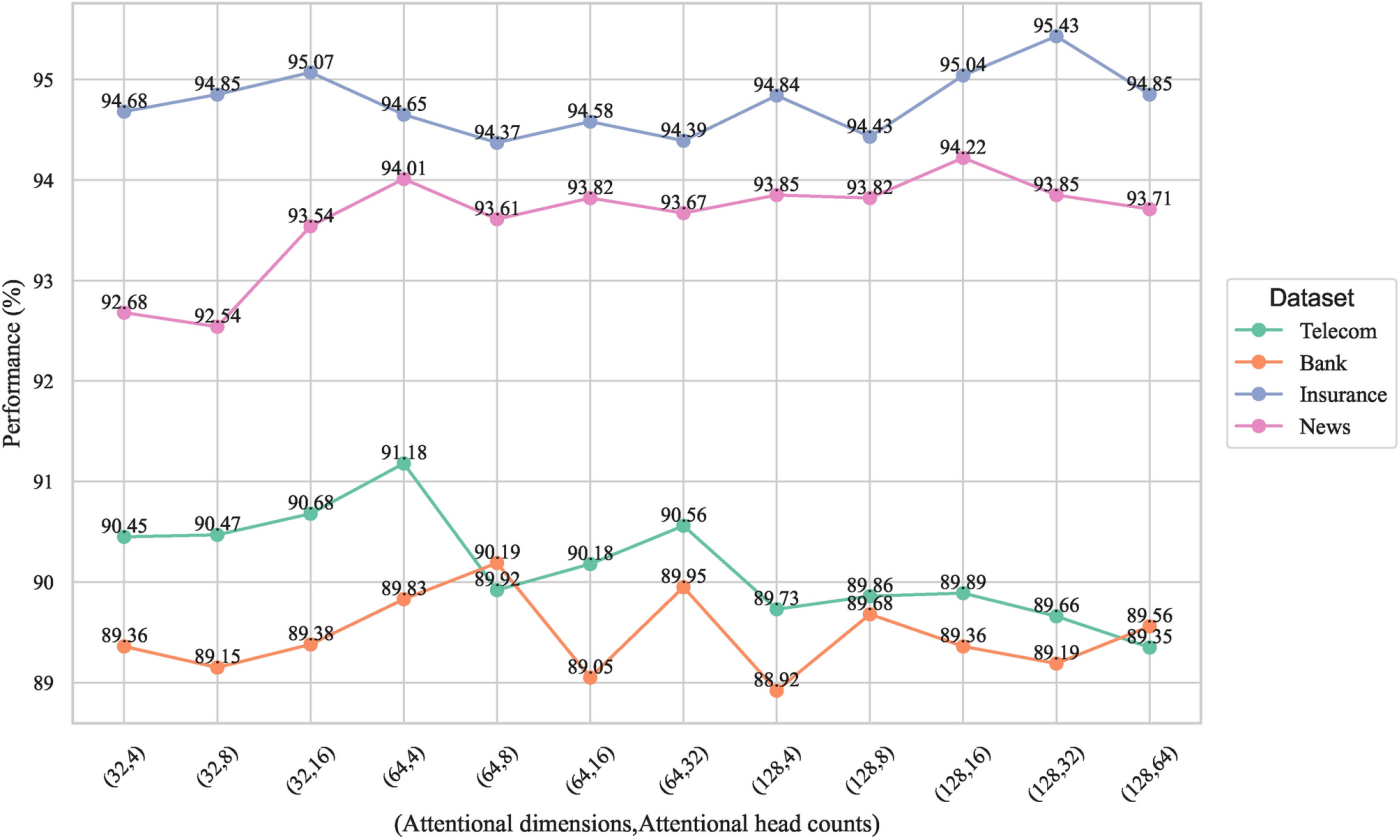
F1 values for different attentional dimensions and attentional head counts.

In this experiment, a systematic grid search was conducted to analyze the key parameters of the CCP-Net model, focusing on two key parameters in Multi-Head Self-Attention: Attentional dimensions and Attentional head counts. In the evaluation process, we chose the F1 value as the main metric to evaluate the performance of the model in the customer churn prediction task under different parameter configurations. The parameter ranges for the grid search are set as follows: Attentional dimensions are 32, 64, and 128, and Attentional head counts are 4, 8, 16, 32, and 64. Figure 12 show the variation in the performance of the CCP-Net model in the four industry datasets (Telecom, Bank, Insurance, News) for different attentional dimensions and attentional head counts.

#### Performance variation analysis

The experimental results show that different datasets exhibit significant performance differences under attentional dimensions and attentional head counts configurations. For example, in the Telecom dataset, the model performance reaches the highest with an F1 value of 91.18% when the attentional dimensions are 64 and attentional head counts are 4, while in the Bank dataset, the attentional dimensions are 64 and attentional head counts are 8, the F1 value reaches 90.19%. For the Insurance dataset, the configuration with attentional dimensions of 128 and attentional head counts of 32 performs the best with an F1 value of 95.43%, while in the News dataset, the configuration with attentional dimensions of 128 and the configuration with attentional head counts of 16 achieved the best results with an F1 value of 94.22%.

These results show that datasets with larger sample sizes (Insurance and News) typically require larger attentional dimensions (128) and more attentional head counts (16 or 32) to capture more features and complex patterns in the data. This suggests that when the data volume increases, the model requires a higher representation capability to extract information efficiently. In contrast, for datasets with relatively small sample sizes (Telecom and Bank), smaller attentional dimensions (64) and fewer attentional head counts (4 or 8) are sufficient to satisfy the model performance requirements and help avoid overfitting.

In summary, we can see that in Multi-Head Self-Attention, the selection of attentional dimensions and attentional head counts should be adjusted according to the characteristics of the dataset, especially when the dataset has a large sample size, the use of larger attentional dimensions and more attentional head counts can significantly improve the model performance. While datasets with smaller sample sizes, smaller attentional dimensions, and attentional head counts can achieve excellent performance.

#### Optimal parameter configuration

Based on the results of the grid search, we determined the optimal configuration of each parameter of the CCP-Net model, as shown in Table 3. With this optimal parameter configuration, the CCP-Net model demonstrates the best performance in the customer churn prediction task, which fully demonstrates the importance of optimal parameter selection to enhance the model’s prediction capability. With this configuration, the model can effectively capture the complex patterns and features in the data, thus achieving excellent classification results on datasets from different industries.

**Table 3:** CCP-Net model parameters.

Model parameter	Dataset			
	Telecom	Bank	Insurance	News
Multi-Head Self-Attention dimension	64	64	128	128
Multi-Head Self-Attention head counts	4	8	32	16
LSTM hidden layer size	100			
LSTM layers	1			
CNN input dimension size	[100,64]		[256,128]	[128,64]
Convolution kernel size	[10,5]			
padding	[5,3]			
pooled kernel size	2			

### Comparative experimental analysis

To validate the performance of the CCP-Net model, a variety of state-of-the-art machine learning and deep learning algorithms commonly used for customer churn prediction were selected for systematic comparison in this study, including SVM, Decision Tree, KNN, XGBoost, Random Forest, MLP, TabNet, CNN, GRU, LSTM, BiGRU, BiLSTM, Transformer, GRU-CNN, LSTM-CNN, BiGRU-CNN, BiLSTM-CNN, LSTM-Attention, and FCLCNN-LSTM. Table 4 summarises the comparative experimental results of each model, and the results show that CCP-Net performs excellently on datasets from four different industries and has obvious advantages in feature extraction and processing complex data. By combining Multi-Head Self-Attention, BiLSTM, and CNN, CCP-Net can capture global and local features efficiently, which makes it more advantageous in processing complex data. In contrast, while single models (e.g., LSTM, GRU) excel in time series data processing, they cannot adequately model global dependencies. At the same time, traditional machine learning methods are highly dependent on feature engineering and are less adaptable.

**Table 4:** Comparison of experimental results.

Model name	Telecom				Bank			
	Accuracy (%)	Precision (%)	Recall (%)	F1 (%)	Accuracy (%)	Precision (%)	Recall (%)	F1 (%)
SVM	84.95	87.43	81.98	84.59	84.69	84.27	85.31	84.78
Decision tree	81.53	81.69	81.68	81.68	80.88	80.12	82.19	81.13
KNN	82.77	83.50	82.07	82.76	83.95	82.67	85.91	84.25
XGBoost	85.38	86.70	83.87	85.25	87.15	86.48	88.07	87.27
Random forest	85.54	87.34	83.45	85.34	87.01	87.02	87.02	87.01
MLP	82.78	83.43	82.38	82.80	83.73	83.65	83.85	83.74
TabNet	85.16	86.57	83.56	85.02	84.95	85.22	84.61	84.89
CNN	88.32	91.88	84.27	87.87	85.80	89.48	81.18	85.11
GRU	88.35	91.13	84.75	87.69	85.92	89.45	81.85	85.58
LSTM	88.43	91.17	85.31	88.06	86.31	89.62	82.17	85.71
BiGRU	88.78	91.53	85.12	88.13	87.02	89.77	82.34	85.91
BiLSTM	88.72	91.61	85.36	88.31	87.23	89.94	83.85	86.74
Transformer	89.48	91.81	86.08	88.85	88.05	90.15	86.32	88.19
GRU-CNN	89.29	91.22	86.18	88.52	87.18	89.68	83.96	86.38
LSTM-CNN	89.51	91.23	86.28	88.79	87.48	89.91	84.34	87.02
BiGRU-CNN	90.57	91.96	89.25	90.53	88.39	90.35	85.72	88.05
BiLSTM-CNN	90.91	92.09	89.66	90.84	88.65	90.82	86.01	88.31
LSTM-Attention	89.85	91.54	86.63	89.07	87.65	90.26	83.63	86.91
FCLCNN-LSTM	90.58	91.88	89.73	90.78	88.84	90.75	88.07	89.17
Attention-BiGRU-CNN	90.95	92.11	89.77	90.86	89.51	90.63	88.12	89.48
CCP-Net	91.17	92.19	90.24	91.18	89.68	91.96	88.41	90.19

Model name	Insurance				News			
	Accuracy (%)	Precision (%)	Recall (%)	F1 (%)	Accuracy (%)	Precision (%)	Recall (%)	F1 (%)
SVM	82.74	81.91	84.09	82.97	84.05	85.43	83.97	84.38
Decision tree	78.11	78.57	77.31	77.92	80.39	80.52	84.69	82.54
KNN	78.18	79.97	75.27	77.52	81.39	79.61	82.49	81.22
XGBoost	85.84	84.39	87.96	86.12	86.84	87.12	87.49	87.30
Random forest	85.31	83.35	88.30	85.73	88.85	88.02	90.79	89.38
MLP	78.42	78.37	78.56	78.44	81.69	80.11	83.49	81.70
TabNet	82.85	81.40	85.27	83.25	82.01	81.19	84.86	82.98
CNN	85.89	88.84	82.16	85.35	88.21	86.78	88.68	87.74
GRU	86.38	87.36	83.49	85.32	88.04	89.58	86.31	87.72
LSTM	86.42	87.56	84.93	86.20	88.12	89.89	86.52	88.11
BiGRU	89.18	90.68	88.69	89.93	90.48	91.35	88.41	90.02
BiLSTM	89.95	90.83	88.75	89.76	90.65	91.62	88.38	90.59
Transformer	90.68	91.35	90.31	90.83	90.79	92.38	89.01	90.67
GRU-CNN	93.24	93.95	92.05	92.74	90.28	91.68	89.85	90.77
LSTM-CNN	93.65	94.25	92.89	93.56	90.82	92.86	89.91	91.36
BiGRU-CNN	93.63	94.05	92.68	93.25	91.65	92.96	90.24	91.62
BiLSTM-CNN	93.96	94.60	93.00	93.77	91.83	93.37	90.61	91.90
LSTM-Attention	93.75	93.23	92.71	92.97	91.42	92.96	90.70	91.81
FCLCNN-LSTM	94.14	94.35	93.58	93.96	92.86	94.28	91.57	92.89
Attention-BiGRU-CNN	94.95	94.89	94.78	94.86	93.12	94.26	92.52	93.48
CCP-Net	95.86	95.87	95.03	95.43	94.08	95.12	93.24	94.22

#### CCP-Net vs Machine learning algorithm

The selected machine learning models (e.g., SVM, Decision Tree, KNN, etc.) rely on complex feature engineering and hyper-parameter tuning, and thus the performance fluctuates widely on different datasets. For example, the choice of regularisation parameters and kernel function for SVM, the tree depth for the Decision Tree, and the number of neighbors K for KNN are all key factors affecting model performance. If these hyperparameters are not reasonably tuned, the model performance may be significantly degraded. For example, on the Insurance dataset, the accuracy of the Decision Tree is only 78.11%. At the same time, its Precision, Recall, and F1 values are 78.57%, 77.31%, and 77.92%, respectively, which are much lower than the CCP-Net model’s values of 95.86%, 95.87%, 95.03%, and 95.43%. As a hybrid neural network, CCP-Net can automatically learn the data representation, significantly reduce the reliance on manual feature engineering, and effectively extract complex features through its internal structure. Compared to algorithms such as KNN which are sensitive to noise and outliers, CCP-Net’s deep structure can better adapt to different data distributions, thus showing greater robustness in dealing with outliers. This design allows CCP-Net to consistently maintain high performance on multiple datasets, whereas traditional machine learning methods often perform poorly in the face of feature fluctuations.

While integrated learning methods (such as XGBoost and Random Forest) offer improved performance compared to a single model, their complexity and computational cost subsequently increase, and the interpretability of the model is often inferior to that of a single model. CCP-Net, through its deep learning structure, provides an approach that balances performance and interpretability. In experiments on four datasets, CCP-Net generally outperforms these machine learning models on all metrics, with increases ranging from 1% to 6%. For example, in the Telecom dataset, CCP-Net’s Precision is 92.19%, while KNN’s Precision is only 83.50%; in the Bank dataset, CCP-Net’s F1 value is 90.19%, much higher than Random Forest’s 87.01%. These results demonstrate the effectiveness of CCP-Net in dealing with complex features.

#### CCP-Net vs Single deep learning algorithm

For single deep learning algorithms such as MLP, TabNet, CNN, GRU, LSTM, BiGRU, BiLSTM, and Transformer, these models may not extract information comprehensively when processing data. For example, MLP has a simple structure containing only input, hidden, and output layers, and lacks a specialized feature extraction structure, leading to its poor performance on various metrics; its Accuracy of 78.42% and F1 of 78.44% on the Insurance dataset are the worst performers among the listed deep learning algorithms. Although TabNet performs well with structured datasets, it does not capture long-term dependencies in time series data as effectively as models specifically designed for time series prediction (e.g., GRU, LSTM), and therefore performs poorly with data containing time series.CNNs typically use fixed-size convolutional kernels, which limits their performance when dealing with data that has variable length or complex ability when dealing with sequence data with variable length or complex temporal dependencies. Although GRU, LSTM, BiGRU, and BiLSTM have memory capabilities when processing sequence data, they may not be able to effectively capture important information in the sequences when processing long sequence data due to the gradient vanishing or explosion problem. In contrast, CCP-Net combines Multi-Head Self-Attention, BiLSTM, and CNN, which fully utilizes the advantages of global and local feature extraction to overcome the gradient problem in long sequence processing, and therefore exhibits stronger prediction ability on diverse features. In addition, the Transformer model’s self-attention mechanism excels in capturing global dependencies but lacks the flexibility to deal with local features, whereas the design of CCP-Net takes both into account, resulting in superior performance in the face of complex time series data.

In the comparison experiments, the CCP-Net model excels in all performance metrics. For example, on the Telecom dataset, the Precision of CCP-Net is 92.19%, while the Precision of LSTM and BiLSTM is 91.17% and 91.61%, respectively. On the News dataset, the F1 value of CCP-Net is 94.22%, while the F1 values of Transformer and CNN are 90.67% and 87.74%, respectively. These results show that CCP-Net can perform effective feature extraction on diverse features through its unique architectural design, demonstrating strong generalization ability.

#### Alternative model analysis

Table 5 shows the performance comparison of GRU, LSTM, BiGRU, and BiLSTM on the Telecom dataset. Although GRU and LSTM are similar in accuracy, LSTM has a slight advantage in Precision and Recall, with an F1-Score of 88.06%, which is 0.37% higher than GRU. This difference indicates that LSTM is better at capturing complex dependencies in long time series; especially when dealing with long-time dependencies, it can effectively avoid the problem of gradient disappearance, thus enhancing the overall performance of the model.

**Table 5:** Alternative model analysis (GRU vs. LSTM).

Model name	Telecom				
	Accuracy (%)	Precision (%)	Recall (%)	F1 (%)	Time(s)
GRU	88.35	91.13	84.75	87.69	16.89
LSTM	88.43	91.17	85.31	88.06	17.25
BiGRU	88.78	91.53	85.12	88.13	17.54
BiLSTM	88.72	91.61	85.36	88.31	17.85
BiGRU-CNN	90.57	91.96	89.25	90.53	18.28
BiLSTM-CNN	90.91	92.09	89.66	90.84	18.87
Attention-BiGRU-CNN	90.95	92.11	89.77	90.86	30.35
CCP-Net	91.17	92.19	90.24	91.18	32.94

In the comparison between BiGRU and BiLSTM, the F1-Score of BiLSTM (88.31%) is slightly higher than that of BiGRU (88.13%). This difference is mainly attributed to the gating mechanism of LSTM, which gives BiLSTM an advantage in modeling long-term dependencies. Although BiGRU is better in terms of computational efficiency, BiLSTM is more suitable for processing complex time series data, especially in capturing long-term dependencies.

After combining with CNN, the F1-Score of BiLSTM-CNN improves to 90.84%, which is higher than the 90.53% of BiGRU-CNN. This result shows that the CNN layer can effectively enhance the local feature extraction and pattern capture of BiLSTM, and further improve the overall performance of the model, especially in the high-dimensional feature space.

By integrating Multi-Head Self-Attention, BiLSTM, and CNN, CCP-Net achieves an F1-Score of 91.18% on the Telecom dataset, outperforming Attention-BiGRU-CNN (90.86%). The Multi-Head Self-Attention module captures global dependencies, BiLSTM handles local dependencies, and the CNN layer further refines features. The combination of the three gives CCP-Net a significant advantage in handling complex features and modeling long and short-term dependencies.

Although the training time of CCP-Net (32.94 seconds) is slightly longer compared to Attention-BiGRU-CNN (30.35 seconds), the increase in this time mainly stems from the BiLSTM module, which significantly improves the expressive power and predictive accuracy of the model. Thus, despite being slightly less computationally efficient, the performance improvement of CCP-Net justifies these additional overheads and shows its unique advantages in complex feature modeling and global dependency capture.

#### Comparative experimental conclusions

Although LSTM-CNN, BiLSTM-CNN, LSTM-Attention, and FCLCNN-LSTM have improved performance compared to a single neural network, CCP-Net combines Multi-Head Self-Attention, BiLSTM, and CNNs to show stronger learning capabilities and obtain better performance results. This combination not only enhances the feature extraction ability of the model but also improves the generalization ability of the model to better cope with data with different characteristics. In Precision, a performance metric, the CCP-Net model generally outperforms other models, with an increase of between 1% and 3%. For example, on the Telecom dataset, the F1 value of CCP-Net is 91.18%, while the F1 values of LSTM-CNN and BiLSTM-CNN are 88.79% and 90.84%, respectively; on the News dataset, the F1 value of CCP-Net is 94.22%, while the F1 values of LSTM-Attention and FCLCNN-LSTM have F1 values of 91.81% and 92.89%, respectively. These results show the obvious advantage of CCP-Net in model prediction performance, especially when dealing with complex features, its multi-level feature fusion capability makes it outperform traditional hybrid models.

#### Error bars and T-tests

**Fig. 13:**
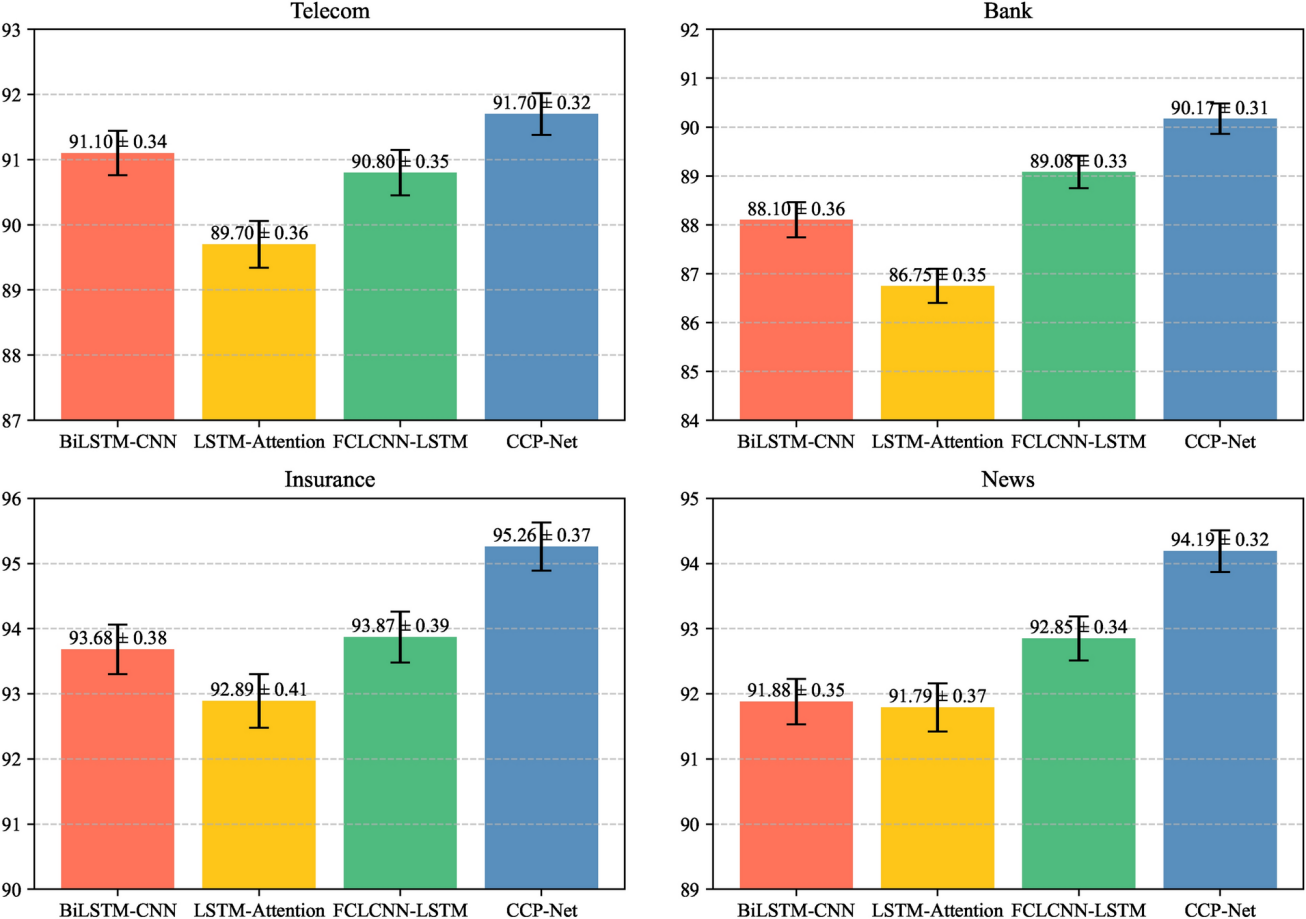
F1 Scores with Confidence Intervals across Different Models and Datasets.

- Error bars
  To analyze the stability and robustness of different hybrid neural network models in more depth, we compare the F1 value performance of BiLSTM-CNN, LSTM-Attention, FCLCNN-LSTM, and CCP-Net on the four datasets of Telecom, Bank, Insurance, and News, and use the error bar to which is visualized (shown in Figure 13). From the results, it can be seen that CCP-Net exhibits the smallest performance fluctuation on all the test datasets, indicating that it has more stability. In particular, in the Bank dataset, the confidence interval of CCP-Net is $90.17 \pm 0.31$, which is significantly smaller than that of other models, further demonstrating its superior stability and robustness. In contrast, BiLSTM-CNN has an F1 value of $88.10\pm 0.36$, LSTM-Attention $86.75\pm 0.35$, and FCLCNN-LSTM $89.08\pm 0.33$, and the performance of these models fluctuates more on the Bank dataset, with a higher width of confidence intervals, suggesting that their performances are relatively unstable, and they may be more stable in different customer churn prediction tasks with greater uncertainty. It can be seen that CCP-Net not only performs well in performance but also demonstrates stronger adaptability to complex tasks through smaller volatility and narrower confidence intervals, which is of great significance for customer churn prediction in practical applications.
- T-tests
  In machine learning and statistical analyses, it is critical to assess the significance of differences in performance between models. The T-test is a commonly used statistical method for comparing the difference between two sample means and determining whether that difference is statistically significant. The T-test enables us to understand whether a model’s performance on a particular task is by chance or reflects an inherent difference in its performance. To further validate the performance improvement of CCP-Net over other hybrid neural network models, we conducted an independent samples T-test to assess the significance of the differences in the F1 values of the models across the datasets. The results of the analysis are shown in Table 6: It can be seen through Table 6 that CCP-Net exhibits a trend of significantly outperforming other hybrid neural network models on all datasets. This indicates that the design and implementation of CCP-Net can effectively enhance the F1 value and thus improve the prediction performance. On the Telecom, Bank, Insurance, and News datasets, the P-values of CCP-Net with other hybrid neural network models (e.g., BiLSTM-CNN, LSTM-Attention, and FCLCNN-LSTM) are all less than 0.05, which demonstrates its significant advantage over other models. These results not only demonstrate the superiority of CCP-Net but also provide a reliable statistical basis for its wide use in practical applications. With the help of a T-test, we confirm the consistency and performance improvement of CCP-Net on multiple datasets, which further proves its effectiveness and reliability in complex tasks such as customer churn prediction.

#### Comparative experimental conclusions

CCP-Net significantly outperforms other models in various performance metrics and demonstrates higher stability and consistency in prediction performance on different industry datasets, reflecting its excellent generalization ability. This generalization capability enables CCP-Net to flexibly adapt to diverse data characteristics and make full use of the synergy between Multi-Head Self-Attention, BiLSTM, and CNN, thus maintaining high prediction accuracy when dealing with complex customer behavior patterns.

**Table 6:** T-test results.

Dataset	Models Comparison	T-statistic	P-value	CCP-Net is superior to this model?
Telecom	CCP-Net vs BiLSTM-CNN	13.18	4.26e-19	yes
	CCP-Net vs LSTM-Attention	17.73	4.29e-25	yes
	CCP-Net vs FCLCNN-LSTM	6.98	3.20e-09	yes
Bank	CCP-Net vs BiLSTM-CNN	37.80	1.47e-42	yes
	CCP-Net vs LSTM-Attention	90.53	3.76e-64	yes
	CCP-Net vs FCLCNN-LSTM	22.43	2.86e-30	yes
Insurance	CCP-Net vs BiLSTM-CNN	30.06	4.58e-37	yes
	CCP-Net vs LSTM-Attention	34.44	2.59e-40	yes
	CCP-Net vs FCLCNN-LSTM	35.49	4.93e-41	yes
Nesw	CCP-Net vs BiLSTM-CNN	27.89	2.70e-35	yes
	CCP-Net vs LSTM-Attention	28.85	4.29e-36	yes
	CCP-Net vs FCLCNN-LSTM	28.67	6.09e-36	yes

The design of CCP-Net takes into account the characteristics of different industries to ensure its broad applicability in multiple domains. For example, in the telecom industry (e.g., Telecom), the global dependency learning capability of the Multi-Head Self-Attention module significantly enhances the capture of complex customer behavioral patterns. CCP-Net achieves an Accuracy of 91.17% on the Telecom dataset, significantly higher than the BiLSTM-CNN’s 90.91% and LSTM-CNN’s 89.51%. This enables CCP-Net to handle large amounts of time-series data and identify potential churn risks promptly. In the financial industry (e.g., Bank and Insurance), the capability of the BiLSTM module in modeling long-term dependencies significantly improves the understanding of the changing dynamics of customer churn. In the Bank dataset, CCP-Net achieves an Accuracy of 89.68%, which is significantly improved compared to BiLSTM’s 87.23%; in the Insurance dataset, CCP-Net’s F1 value of 95.43% outperforms the other algorithms, especially XGBoost’s 86.12% and Random Forest of 85.73%, further validating its effectiveness in capturing customer transaction history and behavioral patterns. In addition, CCP-Net’s feature extraction capability lays the foundation for its application in the media industry (e.g. News), helping companies to deeply analyze users’ reading habits and preferences. In the News dataset, CCP-Net’s Precision reaches 95.12%, much higher than CNN’s 86.78% and BiLSTM’s 91.62%, which enables enterprises to formulate customer retention strategies more effectively.

In the error bar analysis, CCP-Net shows the smallest performance fluctuation on all test datasets, indicating that it has stronger stability. In particular, in the Bank dataset, the confidence interval of CCP-Net is $90.17 \pm 0.31$, which is significantly lower than that of other models, which further demonstrates its superior stability and robustness. In contrast, other models such as BiLSTM-CNN and LSTM-Attention have wider confidence intervals on this dataset, suggesting that their performance is relatively unstable, which may lead to greater uncertainty in practical applications.

To further validate the performance improvement of CCP-Net over other hybrid neural network models, we conducted an independent sample t-test. The results show that in the Telecom, Bank, Insurance, and News datasets, the P-values between CCP-Net and BiLSTM-CNN, LSTM-Attention, and FCLCNN-LSTM are all less than 0.05, indicating significant advantages. For example, in the Bank dataset, CCP-Net vs BiLSTM-CNN has a T-statistic of 37.80 and a P-value of 1.47e-42, showing a significant performance difference. These results provide a solid statistical basis for the effectiveness of CCP-Net in customer churn prediction.

In summary, by comparing with other models, CCP-Net performs well on datasets from different domains, fully proving its effectiveness and reliability in dealing with complex customer churn prediction problems. Overall, CCP-Net not only performs well in the task of customer churn prediction but also it’s flexible structure and powerful feature learning capability provide it with a wide range of application potentials in different industries, thus providing effective customer relationship management solutions for enterprises.

### Ablation experiment analysis

This section provides an in-depth analysis of the importance of each module in the task of customer churn prediction and its impact on model performance through ablation experiments on each module of the CCP-Net model. The results of the ablation experiments are shown in Table 7.

**Table 7:** Results of ablation experiments.

Module			Telecom				Bank			
Attention	BiLSTM	CNN	Accuracy (%)	Precision (%)	Recall (%)	F1 (%)	Accuracy (%)	Precision (%)	Recall (%)	F1 (%)
$\checkmark$	$\times$	$\times$	87.21	91.05	82.78	86.66	84.88	89.12	79.59	84.02
$\times$	$\checkmark$	$\times$	88.72	91.61	85.36	88.31	87.23	89.94	83.85	86.74
$\times$	$\times$	$\checkmark$	88.32	91.88	84.27	87.87	85.80	89.48	81.18	85.11
$\checkmark$	$\checkmark$	$\times$	90.73	92.04	89.31	90.65	87.78	89.69	85.55	87.55
$\checkmark$	$\times$	$\checkmark$	89.32	92.12	86.20	88.93	86.55	89.43	82.93	86.03
$\times$	$\checkmark$	$\checkmark$	90.91	92.09	89.66	90.84	88.65	90.82	86.01	88.31
$\checkmark$	$\checkmark$	$\checkmark$	**91.17**	**92.19**	**90.24**	**91.18**	**89.68**	**91.96**	**88.41**	**90.19**

Module			Insurance				News			
Attention	BiLSTM	CNN	Accuracy (%)	Precision (%)	Recall (%)	F1 (%)	Accuracy (%)	Precision (%)	Recall (%)	F1 (%)
$\checkmark$	$\times$	$\times$	87.67	88.48	86.60	87.51	86.89	84.01	86.59	85.54
$\times$	$\checkmark$	$\times$	89.95	90.83	88.75	89.76	90.65	91.62	88.38	89.90
$\times$	$\times$	$\checkmark$	85.89	88.84	82.16	85.35	88.21	86.78	88.68	87.74
$\checkmark$	$\checkmark$	$\times$	94.44	94.64	94.02	94.28	93.62	93.94	91.68	93.08
$\checkmark$	$\times$	$\checkmark$	93.56	93.74	93.40	93.56	91.31	91.58	91.65	91.60
$\times$	$\checkmark$	$\checkmark$	93.96	94.60	93.00	93.77	91.83	93.37	90.61	91.90
$\checkmark$	$\checkmark$	$\checkmark$	**95.86**	**95.87**	**95.03**	**95.43**	**94.08**	**95.12**	**93.24**	**94.22**

#### Contribution of multi-head self-attention module to model performance

The Multi-Head Self-Attention module plays a crucial role in capturing global dependencies. When this module is removed (the BiLSTM and CNN modules are retained), the overall performance of the model decreases significantly, especially in the extraction of global features. This is specifically shown in:

- In the Telecom dataset, after the removal of multi-head self-attention, accuracy decreased from 91. 17% to 90. 91%, precision decreased from 92. 19% to 92. 09%, recall decreased from 90. 24% to 89. 66%, F1 value decreased from 91. 18% to 90. 84%.
- In the Insurance dataset, removing Multi-Head Self-Attenti reduced Accuracy from 95.86% to 93.96%, Precision from 95.87% to 94.60%, Recall from 95.03% to 93.00%, and F1 value from 95.43% to 93.77%.

These changes suggest that Multi-Head Self-Attention is essential for learning global dependencies, although BiLSTM and CNN can effectively capture time series dependencies and local features. Especially in the Insurance dataset, the module significantly improves the prediction accuracy and enhances the model’s ability to capture complex patterns.

#### Contribution of the BiLSTM module to model performance

The BiLSTM module performs well in time series feature extraction. When the BiLSTM module is removed (the Multi-Head Self-Attention and CNN modules are retained), the model performance decreases significantly, especially in the reduction of Recall and F1 values. The specific performance is shown as follows:

- In the Telecom dataset, after removing BiLSTM, Recall decreases from 90.24% to 86.20% and the F1 value decreases from 91.18% to 88.93%.
- In the Bank dataset, after removing BiLSTM, Recall decreased from 88.41% to 82.93% and the F1 value decreased from 90.19% to 86.03%.

These results show that BiLSTM plays a key role in capturing long-term dependencies of customer behavior, and is particularly good at capturing temporal trends, effectively improving the classification ability of the model.

#### Contribution of CNN module to model performance

The importance of CNN modules in local feature extraction cannot be ignored. The model performance is also significantly affected when removing the CNN module (retaining the Multi-Head Self-Attention and BiLSTM modules). Specifically, it is shown as follows:

- In the Insurance dataset, after removing CNN, Accuracy decreases from 95.86% to 94.44%, Precision decreases from 95.87% to 94.64%, while Recall and F1 values show a corresponding decrease.
- In the News dataset, after removing the CNN, the Recall of the model decreased from 93.24% to 91.68% and the F1 value decreased from 94.22% to 93.08%.

These results show that the CNN module performs particularly well in dealing with localized and nonlinear features, and can significantly improve the overall performance of the model, especially when faced with complex and diverse datasets.

#### Conclusion of ablation experiments

The results of the ablation experiments of the CCP-Net model on different industry datasets show that the complementary functionality of its modules improves the generalization ability of the model. For example, the performance degradation caused by the removal of Multi-Head Self-Attention is particularly noticeable in the Telecom and Insurance datasets, suggesting that this module is indispensable in dealing with industries with complex customer behavior patterns. Meanwhile, BiLSTM is more effective in handling time-series data, which is particularly suitable for the financial sector where long-term dependencies need to be captured. This flexible structural design enables CCP-Net to be widely applied to customer churn prediction tasks in different fields, providing a good basis for its promotion in various industries.

### Impact of ADASYN on the performance of the CCP-Net model

**Fig. 14:**
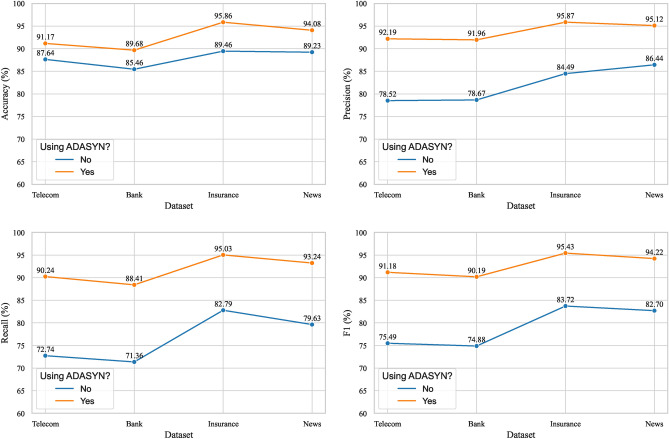
Changes in model performance before and after processing with ADASYN.

To assess the potential impact of ADASYN on the performance of the CCP-Net model, this experiment analyses the performance changes of the CCP-Net model before and after applying ADASYN. The experimental results are shown in Figure 14.

After processing with ADASYN, all performance metrics of the CCP-Net model are significantly improved. For example, in the Telecom dataset, the accuracy of the CCP-Net model is 87.64% using data not processed by ADASYN, and the accuracy of the CCP-Net model improves from 87.64% to 91.17% using data processed by ADASYN; meanwhile, the precision improves from 78. 52% to 92. 19%, the recall improves from 72. 74% to 90. 24%, and F1 improves from 75.49% to 91.18%. Similar trends were observed in other datasets; for example, in the Insurance dataset, accuracy improved from 89. 46% to 95. 86%, precision from 84. 49% to 95. 87%, recall from 82. 79% to 95. 03% and F1 from 83.72% to 95.43%.

This result shows that applying ADASYN as an effective category balancing processing method to the CCP-Net model significantly enhances its overall performance in the customer churn prediction task. This not only demonstrates the effectiveness of ADASYN in dealing with the category imbalance problem but also demonstrates its potential to enhance the model generalization ability and improve the various metrics of the classification task.

### Algorithm complexity analysis

In the task of customer churn prediction, the computational complexity of a model directly affects its training and prediction efficiency, which in turn affects its feasibility and applicability in practical applications. Model complexity not only affects the execution speed but also determines the resource requirements, such as memory and computational power. To better understand the advantages and limitations of CCP-Net, this paper compares it with several other hybrid neural network models (LSTM-CNN, BiLSTM-CNN, LSTM-Attention, FCLCNN-LSTM), analyses its temporal and spatial complexity, and explores the trade-offs between complexity and performance. The specific meanings of the symbols are shown in Table 8:

**Table 8:** Description of the meaning of symbols.

Symbol	Description
*n*	Length of the input sequence
*d*	Dimension of input features
*h*	Dimension of the hidden state
*l*	Number of layers in the LSTM layer
*k*	Size of the convolution kernel
*c*	Number of convolution kernels

#### Time complexity

CCP-Net consists of three modules: Multi-Head Self-Attention, BiLSTM, and CNN. The time complexity for each attention head in Multi-Head Self-Attention to compute the linear transformation of the input features and perform the weighted summation is $O(n \cdot d^2 \cdot h)$, where *h* is the number of attention heads. The computational complexity of BiLSTM in capturing long-term dependencies in the time series is $O(n \cdot h^2 \cdot l)$, where *l* is the length of the sequence. The computational complexity of CNN in performing convolutional operations on the input sequence is: $O(k \cdot n \cdot c)$, where *k* is the filter size, and *c* is the number of channels. Thus, the overall time complexity of CCP-Net is $O(n \cdot d^2 \cdot h + n \cdot h^2 \cdot l + k \cdot n \cdot c)$.

In contrast, the time complexity of LSTM-CNN, BiLSTM-CNN, and FCLCNN-LSTM is: $O(n \cdot h^2 + k \cdot n \cdot c)$, and the time complexity of LSTM-Attention is: $O(n \cdot h^2 + n \cdot d^2)$.

#### Spatial complexity

For CCP-Net, Multi-Head Self-Attention has a space complexity of $O(n \cdot d)$, which is used to store the attention weights and intermediate variables. BiLSTM has a space complexity of $O(n \cdot h \cdot l)$, which is used to store the intermediate variables of the hidden state and the gating unit. CNN has a space complexity of $O(n \cdot c)$, which is used to store the intermediate results of the convolution operation. Therefore, the overall space complexity of CCP-Net is $O(n \cdot d + n \cdot h \cdot l + n \cdot c)$.

In contrast, the space complexity of LSTM-CNN, BiLSTM-CNN, and FCLCNN-LSTM is $O(n \cdot h + n \cdot c)$, and the space complexity of LSTM-Attention is $O(n \cdot h + n \cdot d)$.

#### Time overhead and GPU memory usage

Table 9 shows the computation time and GPU memory usage of different models when running 100 epochs on the Telecom dataset. From the data in the table, it can be seen that there is a significant difference in the time overhead and GPU memory usage of each model during training, which is closely related to the complexity and structural characteristics of the models.

**Table 9:** Model time overhead and GPU memory usage.

Model name	Time(seconds)	GPU memory usage(MB)
LSTM-CNN	18.72	137.31
BiLSTM-CNN	18.85	148.49
LSTM-Attention	27.10	152.66
FCLCNN-LSTM	19.85	142.25
CCP-Net	32.94	168.06

- Time overhead
  The training time of LSTM-CNN and BiLSTM-CNN is 18.72 seconds and 18.85 seconds respectively, which is not much different from each other, reflecting the lower computational requirements of these models, which are suitable for tasks with high response time requirements.
  The training time of LSTM-Attention is significantly longer at 27.10 seconds, which is closely related to the introduction of the Attention mechanism, which requires more computational resources to capture the global dependency information, which increases the computation time of the model.
  The training time of FCLCNN-LSTM is 19.85 seconds, which is slightly higher than that of LSTM-CNN and BiLSTM-CNN, but still within a reasonable range, indicating that the model is also more efficient.
  The training time of CCP-Net is 32.94 seconds, which is the longest among all models. Due to its combination of the Multi-Head Self-Attention, BiLSTM, and CNN modules, the computational requirements are significantly higher. In particular, the introduction of the Attention module and the BiLSTM layer allows the model to handle more complex sequence dependencies and feature interactions, which improves the model’s predictive ability, but at the expense of computational efficiency.
- GPU memory usage
  LSTM-CNN and BiLSTM-CNN show relatively low memory requirements with 137.31 MB and 148.49 MB of GPU memory usage, respectively. This is due to their relatively simple structure, which does not introduce complex global dependency modeling modules (e.g., Multi-Head Self-Attention), and thus is more efficient in terms of memory usage.
  The GPU memory usage of LSTM-Attention is 152.66 MB, which is slightly higher than that of LSTM-CNN and BiLSTM-CNN, which is mainly because the Multi-Head Self-Attention mechanism requires more memory to store the attention weights and intermediate computation results during the training process.
  The memory usage of FCLCNN-LSTM is 142.25 MB, which is slightly higher than that of LSTM-CNN and BiLSTM-CNN, but still lighter compared to LSTM-Attention.
  CCP-Net has the highest GPU memory usage of 168.06 MB, which is closely related to its complex architecture (containing Multi-Head Self-Attention, BiLSTM, and CNN). The Multi-Head Self-Attention module in particular has a significant increase in overhead in terms of memory requirements, resulting in a relatively high memory usage for CCP-Net. Net’s relatively high memory usage.

#### Performance versus complexity trade-offs

In summary, CCP-Net is higher than other hybrid neural network models in both time and space complexity, especially when dealing with complex behavioral patterns, and its ability to capture multi-dimensional features significantly improves prediction accuracy. However, the higher computational resource requirements and training time may cause some limitations in scenarios with high real-time requirements or limited resources. In contrast, models such as LSTM-CNN, BiLSTM-CNN, LSTM-Attention and FCLCNN-LSTM can complete training and prediction in a shorter time due to lower time and space complexity, which is suitable for tasks that require fast response or operate in resource-constrained environments; however, these models are not as good as CCP-LSTM in capturing the global dependency information and handling complex feature interactions are not as good as CCP-Net.

In practice, the selection of an appropriate model should take into account the specific application requirements. If the task emphasizes high performance and complex data processing capability, CCP-Net is a better choice; if it focuses more on real-time performance and resource efficiency, lightweight models (LSTM-CNN or FCLCNN-LSTM) are more suitable. Meanwhile, BiLSTM-CNN and LSTM-Attention can also be used as a compromise between performance and efficiency for scenarios with high requirements for the extraction of temporal features.

## Conclusions and future work

In this paper, we propose a customer churn prediction model, CCP-Net, which integrates Multi-Head Self-Attention, BiLSTM, and CNN. We aim to solve the customer churn problem effectively. To verify the validity of the model, we conducted systematic data preprocessing on datasets from four different industries and implemented ten-fold cross-validation to ensure the reliability of the evaluation results.

After analyzing the experimental parameters, we determine the optimal configuration of each parameter in the CCP-Net model. Through comparative experiments, we can see that CCP-Net outperforms a variety of common deep learning algorithms and hybrid neural networks, demonstrating significant superiority and strong generalization ability. In addition, ablation experiments further validate the architectural soundness of CCP-Net, showing that the combination of Multi-Head Self-Attention, BiLSTM, and CNN endows the model with excellent prediction performance. We also explore the application of ADASYN technology in improving the predictive performance of the model, and the results show that all the performance metrics of CCP-Net are significantly improved after training with ADASYN-treated data, which fully demonstrates the effectiveness of ADASYN in customer churn prediction.

Although CCP-Net performs well in prediction accuracy, its high time and space complexity limits its feasibility in real-time applications. Therefore, we plan to improve the model in future research, working to reduce time and space complexity while maintaining prediction accuracy. Future work will explore more advanced data processing techniques, model optimization methods, and innovative algorithms. We expect that the results of these studies will help organizations to deeply understand and manage their customer relationships, and thus achieve sustainable business development.

## Data Availability

Underlying data supporting the results can be provided by sending a request to the corresponding author.

## Code availability

The code used for this research is publicly available at https://github.com/segujn/CCP-Net.git.
